# GSDMD-mediated pyroptosis: molecular mechanisms, diseases and therapeutic targets

**DOI:** 10.1186/s43556-025-00249-8

**Published:** 2025-02-25

**Authors:** Yujuan Li, Bin Guo

**Affiliations:** 1 Medical College of Optometry and Ophthalmology, Shandong University of Traditional Chinese Medicine, Jinan, 250355 China; 2 Shandong Provincial Key Laboratory of Integrated Traditional Chinese and Western Medicine for Prevention and Therapy of Ocular Diseases, Shandong Academy of Eye Disease Prevention and Therapy, Affiliated Eye Hospital of Shandong University of Traditional Chinese Medicine, Jinan, 250002 China

**Keywords:** Pyroptosis, GSDMD, Cell heterogeneity, Mechanism, Disease, Therapy

## Abstract

Pyroptosis is a regulated form of inflammatory cell death in which Gasdermin D (GSDMD) plays a central role as the key effector molecule. GSDMD-mediated pyroptosis is characterized by complex biological features and considerable heterogeneity in its expression, mechanisms, and functional outcomes across various tissues, cell types, and pathological microenvironments. This heterogeneity is particularly pronounced in inflammation-related diseases and tumors. In the context of inflammatory diseases, GSDMD expression is typically upregulated, and its activation in macrophages, neutrophils, T cells, epithelial cells, and mitochondria triggers both pyroptotic and non-pyroptotic pathways, leading to the release of pro-inflammatory cytokines and exacerbation of tissue damage. However, under certain conditions, GSDMD-mediated pyroptosis may also serve a protective immune function. The expression of GSDMD in tumors is regulated in a more complex manner, where it can either promote immune evasion or, in some instances, induce tumor cell death. As our understanding of GSDMD's role continues to progress, there have been advancements in the development of inhibitors targeting GSDMD-mediated pyroptosis; however, these therapeutic interventions remain in the preclinical phase. This review systematically examines the cellular and molecular complexities of GSDMD-mediated pyroptosis, with a particular emphasis on its roles in inflammation-related diseases and cancer. Furthermore, it underscores the substantial therapeutic potential of GSDMD as a target for precision medicine, highlighting its promising clinical applications.

## Introduction

**Fig. 1:**
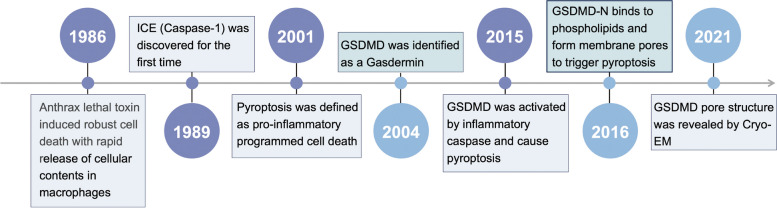
History of pyroptosis. The history of pyroptosis began in 1986 when researchers observed that macrophages infected with anthrax lethal toxin underwent rapid cell membrane rupture and intracellular content release, resulting in cell death [13]. Initially, this phenomenon was thought to involve caspase-1-mediated apoptosis [14]. However, in 2001, it was recognized as distinct from traditional apoptosis, leading to the identification of a novel form of cell death termed pyroptosis [15]. Research on pyroptosis progressed slowly. In 2004, GSDMD was identified as a member of the gasdermin family, although its connection to pyroptosis remained unclear [16]. A major breakthrough occurred in 2015 when the teams of Shao Feng and Vishva M. Dixit independently published studies in *Nature*, establishing pyroptosis as an inflammatory form of cell death mediated by GSDMD [1, 2]. In 2016, it was discovered that pyroptosis involves the binding of N-terminal fragment of GSDMD (GSDMD-N) to phospholipids, leading to pore formation in the cell membrane and initiating pyroptosis [17–20]. Finally, in 2021, cryo-electron microscopy revealed the structural details of GSDMD pores, providing critical insights into the mechanism of pyroptosis [21]

Pyroptosis, a regulated form of inflammatory cell death mediated by Gasdermin proteins, plays a critical role in immune regulation and the progression of various diseases [1–12]. Although first reported in 1986, research on pyroptosis advanced slowly until 2015, when Feng Shao and Vishva M. Dixit’s teams identified GSDMD as the central executor of pyroptosis [1, 2, 13–15]. This breakthrough spurred extensive investigations into the structure, expression patterns, molecular mechanisms, pathological roles, and therapeutic potential of GSDMD (Fig. 1).

GSDMD expression exhibits significant heterogeneity across tissues and cell types. Under resting conditions, GSDMD is highly expressed in tissues such as the small intestine, spleen, and liver but is relatively low in the heart [22]. In non-tumor diseases, GSDMD expression and activity are markedly upregulated, driven by pathological conditions and tissue-specific microenvironments [22–27]. In tumors, its expression is significantly influenced by the tumor microenvironment, which is dynamically regulated by tumor and immune cells [28, 29].

Structurally, GSDMD comprises an GSDMD-N and a C-terminal fragment (GSDMD-C), which form a self-inhibited conformation under normal conditions [30, 31]. Upon stimulation by damage-associated molecular patterns (DAMPs) or pathogen-associated molecular patterns (PAMPs), activated caspase-1 or caspases-4/ 5/ 11 cleave GSDMD, releasing the N-terminal fragment. This fragment oligomerizes and forms pores in the cell membrane, triggering the release of inflammatory cytokines and inducing pyroptotic cell death [1, 2, 32–37]. Beyond pyroptosis, GSDMD is also involved in non-pyroptotic processes such as autophagy, with its activation pathways, cleavage sites, and pore-forming activities being intricately regulated by pathological conditions and microenvironments, influencing its biological functions and inflammatory responses [38].

In various diseases, GSDMD mediates the release of inflammatory cytokines and pyroptosis via the canonical NOD-, LRR- and pyrin domain-containing protein 3 (NLRP3)-caspase-1-GSDMD pathway and the non-canonical caspase-11 pathway, while displaying context-dependent functionality. For example, in acute myocardial infarction (MI), GSDMD exacerbates inflammatory damage by modulating autophagy in neutrophils, whereas in experimental colitis, it enhances mucosal integrity to counter bacterial invasion, providing protective effects [22, 39]. In tumors, GSDMD demonstrates dual roles: it can suppress tumor growth or promote tumor proliferation and migration through immune suppression mechanisms [28, 29]. A comprehensive understanding of the context-dependent mechanisms of GSDMD is crucial for developing targeted therapeutic strategies. Although GSDMD’s roles in immune and inflammatory regulation are well-recognized, its primary effects are often associated with exacerbating organ damage and disease severity. Currently, no approved GSDMD inhibitors exist, emphasizing the need for the development of effective targeted inhibitors with significant clinical potential.

In conclusion, this review summarizes the differential expression, tissue- and cell-specific mechanisms, and diverse roles of GSDMD in both resting and pathological states. It further explores the potential applications of targeting GSDMD in drug development and precision medicine, providing critical insights for future research.

## Cellular Heterogeneity in GSDMD Expression

The expression of GSDMD protein exhibits significant differences between resting and pathological states. In the resting state, GSDMD expression is regulated by its distribution across various cell types and tissues, leading to substantial variation in expression levels between different tissues. Under pathological conditions, GSDMD expression undergoes notable changes compared to the resting state, though the patterns of alteration are inconsistent between non-tumor and tumor-associated diseases. In non-tumor diseases, GSDMD expression is notably upregulated, while in tumor-related diseases, the changes in expression are more complex. Nevertheless, in both contexts, alterations in GSDMD protein expression are closely associated with disease pathogenesis and the changes in the pathological microenvironment.

### Variations in GSDMD expression across tissues and cells in resting states

In the resting state, GSDMD expression levels vary significantly across different tissues, primarily due to cellular heterogeneity within these tissues. The expression of GSDMD is closely related to the cellular composition of each tissue. For instance, GSDMD expression is higher in the small intestine and liver, while lower levels are observed in tissues such as the heart [22]. In the small intestine, major cell types such as epithelial cells, endothelial cells, and mesenchymal cells exhibit high levels of GSDMD expression, which collectively contribute to the elevated overall expression of GSDMD in this tissue [40, 41]. In the liver, approximately 60% of the cells are hepatocytes, which express high levels of GSDMD. Other key cell types, such as endothelial cells, bile duct cells, hepatic stellate cells, and immune cells, also express GSDMD at varying levels, contributing to the overall high expression of GSDMD in the liver [42, 43]. In contrast, GSDMD expression is relatively low in the heart, likely due to the fact that cardiomyocytes, which constitute 20%—35% of heart tissue, exhibit low levels of GSDMD expression, resulting in lower overall expression in heart tissue [5, 44–48].

Additionally, GSDMD expression exhibits spatial variation within the same tissues, and expression levels can vary for the same cell type across different tissues. For example, in the small intestine, GSDMD expression ranges from 201.7 to 375.4 in the proximal region, with an average expression level of 80 to 161.9 across the entire small intestine, as reported by The Human Protein Atlas. In macrophages, GSDMD expression also varies by tissue, with levels of 58.8, 94.7, and 43.4—93.0 in bone marrow, kidneys, and lungs, respectively (data from The Human Protein Atlas). These differences may be attributed to the presence of distinct macrophage subtypes [40, 49–51]. However, the specific roles and mechanisms underlying these expression differences remain unexplored.

**Fig. 2:**
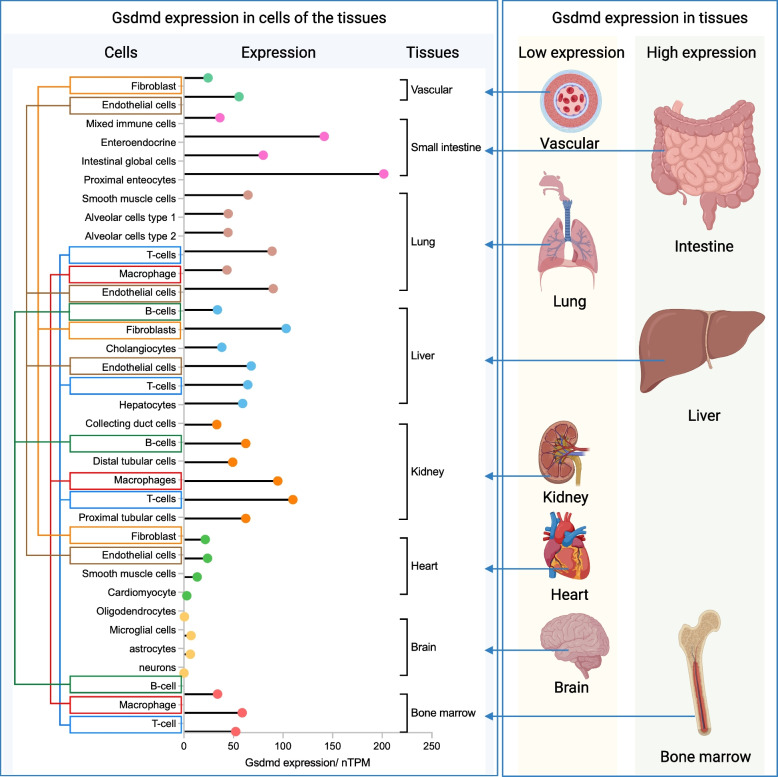
Expression of GSDMD in different tissues and cells. GSDMD is highly expressed in the intestine, liver, and bone marrow, while its expression is low in blood vessels, lung, kidney, heart, and brain. This expression pattern correlates with the levels of GSDMD in specific cells within these tissues. The Human Protein Atlas database provides quantitative data on GSDMD expression at the single-cell level across various tissues

In conclusion, although numerous studies have examined GSDMD expression under resting conditions, the specific mechanisms driving its differential expression across tissues and cell types remain inadequately understood and warrant further investigation [52] **(**Fig. 2**)**.

### Alterations in GSDMD expression across tissues and cells in disease conditions

GSDMD expression is significantly upregulated in non-tumor diseases (non-infectious, infectious and autoimmune diseases) and significantly change in tumor diseases.

In non-tumor diseases, this increased expression occurs in multiple tissues, including the heart, liver, brain, spleen, small intestine and kidney, involving both parenchymal and immune cells. Parenchymal cells include hepatocytes and cardiomyocytes, while immune cells consist of bone marrow- and spleen-derived macrophages and neutrophils, as well as tissue-specific immune cells such as microglia in the central nervous system [22–27, 53]. In heart and liver diseases, GSDMD expression is markedly elevated in cardiomyocytes and hepatocytes, contributing to the regulation of pathological processes. For example, in heart diseases, myocardial injury induces oxidative stress in cardiomyocytes, leading to increased GSDMD expression. Activated caspase-11 cleaves GSDMD, forming GSDMD-N pores that release interleukin-18 (IL-18) and promote cardiomyocyte pyroptosis, thereby exacerbating reperfusion-induced heart damage [54]. Similarly, in alcoholic hepatitis, alcoholic/ non-alcoholic fatty liver disease, and hepatic ischemia–reperfusion injury, GSDMD expression is significantly upregulated in hepatocytes. Conditional knockout of GSDMD in hepatocytes effectively reduce inflammatory cell infiltration and fibrosis in these diseases [55–60]. As a key mediator of inflammatory programmed necrosis, GSDMD plays an important role not only in circulating immune cells in the bone marrow but also in tissue-resident immune cells. In MI, GSDMD expression increases as neutrophils accumulate in large numbers, mediating GSDMD expression in the bone marrow. Activated neutrophils secrete interleukin-1β (IL-1β) and promote granule formation [22, 61]. In cerebral ischemia and neurodegenerative diseases, cytosolic double-stranded DNA (dsDNA) activates the cyclic GMP-AMP synthase (cGAS) signaling pathway in microglia, promoting the expression of (Absent in melanoma 2) AIM-2/ NLRP3 and GSDMD, which leads to cell polarization and pyroptosis [26, 27, 62]. In conclusion, GSDMD expression is upregulated under pathological conditions and exhibits cell-type-specific functions **(**Table 1**)**.

**Table 1:** The change of GSDMD expression in non-tumor disease

Classic	Diseases		Up of the organ	Cell types	References
Non-infectious diseases	Heart diseases	MI	Heart	Bone marrow derived neutrophil	[22, 23]
		Atherosclerosis	Aortic root	Myeloid cell, endothelial cell	[24, 25]
		MI/ R	Heart	Cardiomyocyte	[54]
		Diabetic cardiomyopathy	Heart	Cardiomyocyte	[63]
	Liver diseases	Acute and chronic liver disease	–	iNKT cell	[64]
		Alcoholic hepatitis	Liver	Hepatocyte	[55]
		Alcoholic steatohepatitis	Liver	Hepatocyte	[56]
		Liver warm ischemia–reperfusion injury	Liver	Macrophage	[65]
		Non-alcoholic steatohepatitis	Liver	Hepatocyte	[57–59]
		Noninfectious liver injury	Liver	Hepatocyte	[60]
		Obesity-associated hepatocellular carcinoma	Liver	Hepatic stellate cell	[66]
	Nervous diseases	Alzheimer’s disease	Brain	Oligodendrocyte	[67]
		ALS	Brain	White matter Microglia	[53]
		Brain injury after subarachnoid haemorrhage	Brain (cerebrospinal fluid)	Primary cortical neuron	[68]
		Ischemic stroke	Brain	Microglia	[26, 27]
		PD	Brain	Microglia	[62]
		Vascular dementia	Brain	–	[69]
	Others	Chronic kidney disease	Kidney	Podocyte	[70]
		Acute kidney injury	Kidney	Renal tubular epithelial	[71]
		Age-related macular degeneration	Eye	Retinal pigmented epithelium	[72]
		CRSwNP	Nose (nasal mucosa)	–	[73]
Infectious diseases	DFUs		Foot	Keratinocyte	[74]
	Endotoxemia-induced lung injury		Lung	Endotheail	[75]
	Malaria		Brain	Dendritic cell	[76]
	Rhinovirus infection		Nasal mucosa	Epithelial	[77]
	Sepsis		Bone marrow	Macrophage	[78–80]
	Shock		Small intestine	Endothelial/ macrophage	[81]
	Brain infection		Brain	Microglia	[82]
Autoimmune diseases	Experimental autoimmune eTNFncephalomyelitis		Bone marrow	Peripheral myeloid cell	[83]
	FMF		Spleen	Monocyte/ macrophage	[84]
	IBD		Intestinal	Epithelial cell, macrophage	[39, 85–87]
	Kawasaki disease		Vascular	Endothelial cell	[88]
	Multiple sclerosis		Central nervous system	Myeloid cell (macrophages/ microglia)	[89]

*MI/ R* Myocardial ischemia–reperfusion, *iNKT* Invariant natural killer T, *ALS* Amyotrophic lateral sclerosis, *PD* Parkinson's disease, *CRSwNP* Chronic rhinosinusitis with nasal polyps, *DFUs* Diabetic foot ulcers, *FMF* Familial mediterranean fever, *IBD* Inflammatory bowel diseases

In tumor diseases, the expression of GSDMD in tumor tissues is closely associated with the tumor microenvironment and primarily regulated by tumor cells and immune cells. In acute inflammatory microenvironments, low GSDMD expression in both tumor and immune cells facilitates tumor growth. Conversely, in chronic inflammatory microenvironments, GSDMD-mediated pyroptosis in immune cells triggers inflammatory responses that further promote tumor progression. The expression of GSDMD in tumor tissues is dynamically controlled by the interactions between tumor cells and immune cells [90].

## GSDMD-mediated signaling pathways

Under DAMPs or PAMPs stimulation, GSDMD undergoes a conformational change, leading to cleavage, pore formation, release of inflammatory cytokines, and pyroptosis. However, due to differences in stimuli and cell types, the GSDMD signaling pathway exhibits distinct characteristics in macrophages, neutrophils, T cells, epithelial cells, and mitochondria.

### GSDMD-mediated signaling pathways in structural-level

GSDMD is composed of two main domains: the GSDMD-N and GSDMD-C, which are connected by a flexible linker peptide. The N-terminal domain contains three α-helices and ten β-sheets, while the C-terminal domain comprises nine α-helices and an antiparallel triplet β-sheet structure [30, 31]. Conformational changes in both the N-terminal and C-terminal domains are involved in auto-inhibition, cleavage, and the formation of pyroptotic pores in the membrane.

**Fig. 3:**
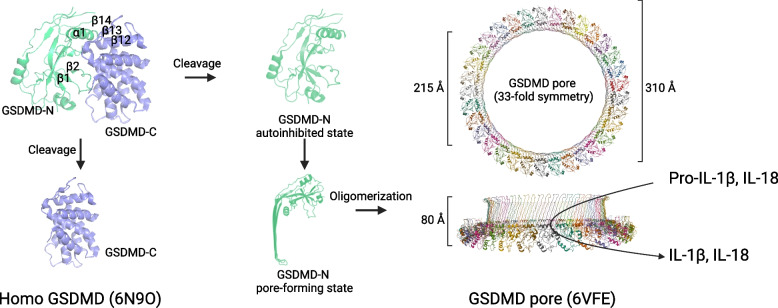
Schematic representation of the structural changes in GSDMD during pyroptosis, illustrating the processes of auto-inhibition, cleavage, and pore-formation. In the autoinhibited state, GSDMD is cleaved into GSDMD-N and GSDMD-C in response to inflammatory stimuli. The GSDMD-N fragment then undergoes conformational changes, enabling its assembly into membrane pores. *Note:* GSDMD auto-inhibition structure (PDB: 6N9O) [11], pore formation structure (PDB: 6VFE) [30]

In the resting state, the β12—β14—β13 sequence of the GSDMD-C domain covers the α1 and α4 helices of the GSDMD-N domain, maintaining the autoinhibitory activity of the full-length GSDMD protein [17]. Upon inflammatory stimulation, this autoinhibitory structure is disrupted, resulting in cleavage at specific linker sites and the subsequent release of GSDMD-C and GSDMD-N [17, 31]. Guided by the α1 and α4 helices, GSDMD-N localizes to the plasma membrane, where it interacts with inner leaflet acidic phospholipids, undergoes conformational rearrangement, and assembles into pores. These pores are composed of 31—34 subunits, with an outer diameter of approximately 30 nm and an inner diameter of roughly 20 nm, thereby initiating pyroptosis [1, 2, 21, 32, 36, 37, 91–98]. However, the precise function of the β1—β2 loop in GSDMD-N during pore formation and the mechanism by which it is masked by GSDMD-C for auto-inhibition remain poorly understood [99] **(**Fig. 3**)**.

### GSDMD-mediated signaling pathways in cellular-level

GSDMD operates through distinct mechanisms in various cell types (macrophages, neutrophils, T cell, epithelial cells and mitochondria) in response to stimuli such as pathogens and food antigens. These processes maybe include the activation and cleavage of GSDMD, GSDMD accumulation and formation of pores in the cell membrane, GSDMD pore-mediated secretion of inflammatory factors and the pyroptosis pathway [100]. Besides, GSDMD can induce the non-pyroptotic pathway.

#### Activation and cleavage of GSDMD

GSDMD activity mainly depends on the cleavage of GSDMD. The cleavage pattern and sites vary with the cell type, stimulating factor and pathological factors.

##### Macrophages

**Fig. 4:**
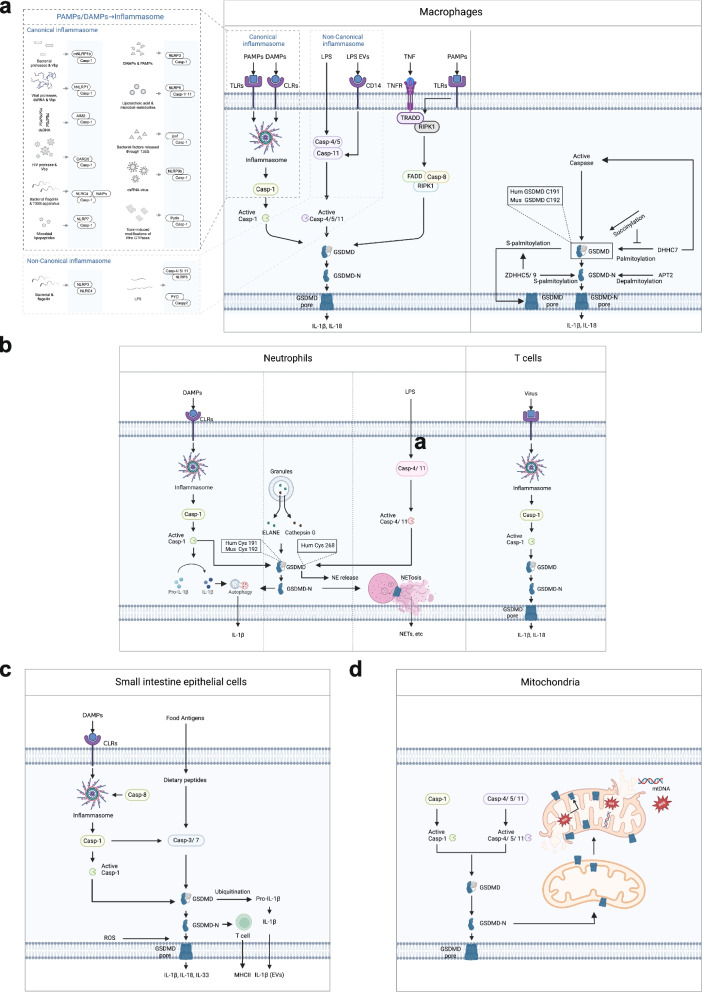
Pyroptosis signaling pathway in various cells. **a**. Macrophage-mediated pyroptosis can be classified into the caspase-1-mediated canonical pathway and the caspase-4/ 11-mediated non-canonical pathway, depending on whether DAMPs or PAMPs are recognized by membrane receptors. Additionally, caspase-8 can mediate pyroptosis in macrophages. The GSDMD-mediated pyroptosis process involves several post-translational modifications, including GSDMD succinylation and palmitoylati. **b.** When neutrophils are stimulated by inflammation, caspase-1 is activated, promoting the cleavage of GSDMD at Cys 191/ 192 and the processing of IL-1β. Granules also cleave GSDMD at Cys 268, facilitating subsequent GSDMD cleavage and IL-1β release. Although IL-1β release depends on GSDMD, it does not pass through GSDMD pores. LPS activates caspase-4/ 11, leading to GSDMD cleavage and subsequent NETosis in neutrophils. In T cells, viral activation and LPS stimulation of the caspase-1 signaling pathway mediate GSDMD cleavage and IL-1β release. **c.** In small intestinal epithelial cells, DAMPs are recognized by membrane receptors, activating the caspase-1 signaling pathway. Caspase-8 expression can trigger inflammasome formation, and IL-1β secretion. Caspase-1 directly activates GSDMD and caspase-7. Food antigens activate the caspase-3/ 7 signaling pathway, mediating GSDMD cleavage and MHCII release. GSDMD can also form pores in the cell membrane to secrete IL-33 independently of caspase-1/ 11. Additionally, increased ROS promotes the accumulation of GSDMD-N on the membrane, and GSDMD mediates the ubiquitination and secretion of pro-IL-1β. **d.** In mitochondria, In the cytoplasm, the cleavage of GSDMD generates GSDMD-N, which forms pores in both the inner and outer mitochondrial membranes, facilitating the release of mtROS and mtDNA into the extracellular space. mtROS also mediate the formation of GSDMD-induced pores in the membranes

In macrophages, GSDMD could be cleaved by caspase-1/ 8/ 4/ 5/ 11 and to form GSDMD-N. Caspase-1 mediated pathway was regarded as the canonical pathway, which is that when lipopolysaccharide (LPS) or DAMPs are recognized by PRRs (pattern recognition receptors), the caspase-1 is activated, promoting NLRP3 [32, 77, 101–115], Nucleotide-binding domain leucine-rich repeat pyrin domain containing 9b (NLRP9b) [116], Nucleotide-binding domain leucine-rich repeat CARD Domain Containing 4 (NLRC4) [117–121], AIM2 [24, 122–124], Pyrin [125, 126], Caspase recruitment domain-containing protein 8 (CARD8) [127–129], Nucleotide-binding domain leucine-rich repeat pyrin domain containing 7 (NLRP7) [130], human nucleotide-binding domain leucine-rich repeat pyrin domain-containing 1 (hNLRP1) [131], mouse nucleotide-binding domain leucine-rich repeat pyrin domain-containing 1b (mNLRP1b) [132, 133] and other inflammasomes. The expression and assembly of these inflammasomes cleave caspase-1, which then mediates the cleavage of GSDMD to form GSDMD-N. Caspase-4/ 5/ 11 pathway was regarded as non-canonical pathway, which is that when PAMPs do not require recognition by PRRs, they can directly activate caspases-4, 5, and 11 to regulate the cleavage of GSDMD [2, 105, 115, 134–141]. Additionally, studies have found that caspase-8 can promote the formation of the Fas-associating protein with a novel death domain (FADD)- Receptor interacting serine/ threonine-protein kinase 1 (RIPK1)-caspase-8 complex and the cleavage of GSDMD under the stimulation of *Yersinia* or LPS [33–35, 142–144]. Caspases-1, 4, 5, and 11 cleave aspartic acid at site 275 of human GSDMD (272FLTD275) or aspartic acid at site 276 of mouse GSDMD (273LLSD276) to form GSDMD-N and GSDMD-C [1, 2, 32, 36, 37]. Caspase-8 cleaved GSDMD at aspartic acid site 276, forming GSDMD-N and GSDMD-C [33, 34] (Fig. 4a).

##### Neutrophils

In neutrophils, stimulation by DAMPs activates the NLRP3-caspase-1 signaling pathway, leading to the cleavage of GSDMD and pro-IL-1β, resulting in the production of GSDMD-N and IL-1β. GSDMD-N does not localize to the plasma membrane (PM) nor does it increase PM permeability or induce pyroptosis. Instead, it predominantly associates with azurophilic granules and LC3^+^ autophagosomes, thereby facilitating the release of IL-1β [38]. When neutrophils are stimulated by LPS or Gram-negative bacteria, caspase-4/ 11 activation induces pyroptosis, leading to the release of neutrophil extracellular traps (NETs) [145–148].

In addition to the caspase-mediated GSDMD activation pathway, neutrophils also utilize neutrophil elastase (ELANE), which is specifically expressed in neutrophils, to independently activate GSDMD. ELANE cleaves GSDMD at a site upstream of the caspase cleavage site, generating a fully active GSDMD-N fragment [37]. The trafficking of GSDMD-N to azurophilic granules induces the leakage of neutrophil elastase (NE) into the cytoplasm, which subsequently leads to secondary cleavage of GSDMD, producing another active form of GSDMD-N [38]. Additionally, Cathepsin G can recognize the Cysteine (Cys) 268 site on GSDMD, cleaving it to produce GSDMD-N, thereby further promoting the release of mature IL-1β and amplifying the inflammatory response [37, 96, 148, 149] (Fig. 4b).

##### T cells

In T cells, caspase-1 is activated by the NLRP3 inflammasome in response to Human Immunodeficiency Virus (HIV) viral DNA and lentiviral short hairpin RNA [108, 110]. Additionally, caspase-1 activation can be mediated by the Caspase recruitment domain-containing protein 8 (CARD8) inflammasome, which is triggered by intracellular HIV-1 protease activity. In this pathway, dipeptidyl peptidase 9 (DPP9) functions as a regulator that inhibits CARD8 activation. Once activated, caspase-1 cleaves GSDMD at Aspartic acid (Asp) 275 in primary human CD4 and CD8 T cells [129, 150] (Fig. 4b).

##### Epithelial cells

In intestinal epithelial cell, GSDMD is cleaved by both caspase-1 and caspase-3/ 7 in small intestinal epithelial cells. In the caspase-1 cleavage pathway, following rotavirus infection, the NLRP9b protein, which is specifically expressed in intestinal epithelial cells, is activated. The RNA helicase DExH-box helicase 9 (Dhx9) recognizes shorter double-stranded RNA (dsRNA) fragments, and together with the adapter protein Apoptosis-associated speck-like protein containing a CARD (ASC) and caspase-1, forms inflammasome complexes that promote GSDMD cleavage [116]. Caspase-8 (C362S) expression can further trigger the formation of ASC puncta and activate caspase-1 [151]. Besides, caspase-1 not only directly activates GSDMD but also activates caspase-7 [152]. In the caspase-3/ 7 cleavage pathway, the D88 site of GSDMD can be cleaved by caspase-3/ 7, forming an approximately 13 kD GSDMD-N terminus and a 42 kD GSDMD-C terminus [100]. GSDMD could be cleaved into the GSDMD p40 fragment by allergen stimulation [153] (Fig. 4c). In brain endothelial cells, the destruction of the blood–brain barrier caused by LPS stimulation is primarily due to the activation of the caspase-4/ 11-GSDMD pathway in brain endothelial cells. Activated GSDMD forms pores in the cell membrane of brain endothelial cells, increasing membrane permeability and inducing pyroptosis [154].

##### Mitochondria

When phagocytosed bacterial endotoxin LPS activates GSDMD, it forms pores on mitochondria, leading to the release of mitochondrial DNA (mtDNA). The leaked mtDNA enters the cytoplasm, triggering inflammasome assembly, caspase-1 activation, and pyroptosis. This process is regulated by MRE11A [114, 155]. In macrophages carrying the Lrrk2G2019S mutation, inflammasome activation increases mitochondrial reactive oxygen species (mtROS) levels, promoting the binding of the pore-forming protein GSDMD to the mitochondrial membrane [156]. Upon stimulation by LPS and Nigericin, GSDMD is cleaved through pyroptotic pathways mediated by caspase-1 and caspase-4/ 5/ 11, generating GSDMD-N. Cytoplasmic GSDMD-N forms pores on both the inner and outer mitochondrial membranes, resulting in reduced mitochondrial abundance, decreased mtROS levels, and the release of proteins and DNA from the mitochondrial matrix and intermembrane space [157] (Fig. 4d).

#### Accumulation and pore formation of GSDMD in membranes

In macrophages, following cleavage, GSDMD-N rapidly accumulates at the cell membrane, selectively targeting specific membrane components. GSDMD-N exhibits a high affinity for phosphoinositides and cardiolipin located on the inner leaflet of the membrane, where it forms pyroptotic pores [1, 17–19]. The formation of GSDMD pores requires the involvement of specific proteins. A forward genetic screen conducted by Charles L. Evavold et al. identified the Ragulator-Rag complex as essential for GSDMD pore formation in macrophages. Besides, mitochondrial poisons that enhance reactive oxygen species (ROS) production can restore GSDMD oligomerization and pore formation [158]. Importantly, due to its lipid-binding preferences, GSDMD-N kills from within the cell, but does not harm neighboring mammalian cells when it is released during pyroptosis [18].

The post-translational modification of GSDMD is a crucial post-translational modification that dictates GSDMD membrane localization and regulates the pyroptosis process. For example, the palmitoyl acyltransferase DHHC7 palmitoylates GSDMD, facilitating its cleavage by caspases. Following this, the palmitoylation of GSDMD-N enhances its transport to the plasma membrane, where APT2 depalmitoylates GSDMD-N, exposing the Cys 192 residue and promoting its oligomerization. Zinc Finger DHHC-Type Palmitoyltransferase 5 (ZDHHC5) and Zinc Finger DHHC-Type Palmitoyltransferase 9 (ZDHHC9)-mediated palmitoylation of GSDMD increases GSDMD-N's affinity for phosphatidylinositol and cardiolipin, regulates its localization on the plasma membrane, and affects its oligomerization [159–161]. S-palmitoylation of GSDMD at Cysteine (Cys) 191 is essential for the formation of GSDMD-N pores. However, S-palmitoylation does not affect GSDMD cleavage and can be enhanced by mitochondria-derived ROS. S-palmitoylation can modify full-length GSDMD, inducing lipid leakage and the formation of pores similar to GSDMD-N. This finding challenges the prevailing notion that GSDMD cleavage is the sole trigger for GSDMD activation [162]. The Cys 191/ Cys 192 site of GSDMD can also undergo succinylation, which negatively regulates the palmitoylation of GSDMD during the pyroptosis process [160].

In addition, in macrophages, proteomic studies have shown that protein phosphatase 1 (PP1) co-localizes with GSDMD, and phosphorylation at sites such as Threonine (Thr) 213 may alter GSDMD pore formation [163]. Succinic acid can also modify GSDMD at Cys 77, impacting its processing levels [18, 164, 165]. Lysosome-derived ROS, released by the Ragulator-Rag complex, can directly modify Cys 192 on GSDMD, affecting its oligomerization and pore formation and ultimately leading to pyroptotic cell death [166]. In small intestinal epithelial cells, the N-terminus can be transported to the nucleus and induce MHCII molecule transcription in intestinal epithelial cells in the upper small intestine, thereby inducing a protective immune response against pathogens [100].

#### Release of inflammatory cytokines via GSDMD pores

GSDMD forms pores in the membrane that mediate the release of inflammatory cytokines. In macrophages, these pores enable the secretion of mature IL-1β, IL-18, and other inflammatory factors, thereby initiating subsequent inflammatory responses [1, 17–19]. The GSDMD-N pores specifically facilitate the secretion of mature IL-1β into the extracellular space. This is primarily due to the fact that GSDMD-N pores, which are negatively charged, allow the passage of positively charged small proteins like IL-1β, whereas negatively charged unprocessed IL-1β or proteins of similar molecular weight are less likely to pass through the pores [98, 167]. Furthermore, the GSDMD-N forms pores with an internal diameter of approximately 10—16 nm in the membrane, allowing small molecules such as IL-1β/ IL-18 (approximately 7.5 nm in diameter) to pass through [17]. The mechanism of IL-1β release in neutrophils and small intestinal epithelial cells, differs from that in macrophages and dendritic cells. Although GSDMD is essential for the release of IL-1β from neutrophils and small intestinal epithelial cells, it cannot form pores on the membrane, preventing IL-1β from being released extracellularly through pyroptotic pores. Studies have found that IL-1β in neutrophils is released through the autophagy pathway [38]. In small intestinal epithelial cells, IL-1β is secreted outside the membrane in the form of exosome cargo [151]. GSDMD can also mediate the ubiquitination of pro-IL-1β, promoting the packaging of IL-1β into secretory vesicles and releasing it extracellularly in a hole-independent manner [85, 168].

In addition to the release of IL-1β, GSDMD mediates cytosolic LPS sensing by caspase-11 triggers the systemic release of galectin-1 via GSDMD pores directly in macrophages [135]. In epithelial cells and hepatic stellate cells, GSDMD is cleaved into the GSDMD p40 fragment, forming pores on the cell membrane and allowing the secretion of IL-33. However, this process of GSDMD cleavage is not dependent on caspase-1/ 11 [66, 153]. In addition to pyroptosis, GSDMD can also mediate non-pyroptotic effects in mitochondria. When the inflammasome is activated in Lrrk2G2019S macrophages, elevated mtROS guides GSDMD to associate with the mitochondrial membrane. Mitochondrial GSDMD pore formation releases mtROS and promotes Receptor-interacting serine/ threonine-protein kinase 1 (RIPK1)/ Receptor-interacting serine/ threonine-protein kinase 1 (RIPK3)/ mixed lineage kinase domain-like protein (MLKL)-dependent necrotic transition [156]. The aggregation of GSDMD in azurophilic granules results in the release of elastase NE into the cytoplasm of neutrophils, which in turn triggers secondary cleavage of GSDMD [38]. GSDMD in neutrophils also affects the formation of neutrophil extracellular traps (NETs). During NETosis, GSDMD is hydrolytically activated by neutrophil proteases, affecting protease activation and nuclear expansion in a feed-forward cycle [146]. Besides, HMGB1 could be released was not via the GSDMD pore, while it is GSDMD depend [169]. 11,12-epoxyeicosatrienoic acid (11,12-EET) as a bioactive, pro-healing oxylipin that is secreted from hyperactive macrophages in a GSDMD-dependent manner [170].

#### Pyroptosis mediated by GSDMD pores

GSDMD pore could mediate pyroptosis ultimately. In macrophages, GSDMD pore induces membrane blebbing and swelling, followed by a loss of cell membrane integrity due to changes in osmotic pressure, ultimately triggering pyroptosis, along with the passive release of lactate dehydrogenase (LDH) [1, 17–19, 171]. GSDMD-mediated plasma membrane rupture is not driven by osmotic pressure but is actively regulated by the protein Ninjurin-1 (NINJ1). In eukaryotic cells, during lytic cell death, the extracellular α-helices of NINJ1 insert into the plasma membrane, promoting the polymerization of NINJ1 monomers into amphipathic filaments that cause membrane rupture [172]. In macrophage-mediated pyroptosis models, forward genetic screening has identified the crucial role of NINJ1 in plasma membrane rupture. In the absence of NINJ1, pyroptosis is inhibited, yet the cell eventually dies without membrane rupture. Consequently, cytosolic components such as LDH are not released into the extracellular environment [173–175].

GSDMD-mediated pyroptosis can be counteracted by certain proteins that facilitate membrane repair. For example, in small intestinal epithelial cells, caspase-7 modulates GSDMD pore formation and preserves cell integrity by cleaving and activating acid sphingomyelinase (ASM), leading to the production of ceramide, which supports membrane repair [152]. In addition, the calcium influx through GSDMD pores acts as a signal for cells to initiate membrane repair by recruiting the endosomal sorting complexes required for transport (ESCRT) machinery to damaged membrane sites, such as the plasma membrane. Inhibition of the ESCRT-III machinery significantly amplifies pyroptosis and enhances interleukin-1β release in both human and murine cells following canonical or noncanonical inflammasome activation [176, 177].

## Heterogeneity of GSDMD function in diseases

GSDMD, a key regulator of pyroptosis, is widely expressed in both non-tumor and tumor cells, playing a role in mediating inflammatory damage in non-tumor diseases and contributing to diverse pathological processes in tumor diseases, including tumor progression and suppression. In non-tumor diseases, GSDMD predominantly exacerbates inflammation and drives disease progression by facilitating the release of inflammatory cytokines. In contrast, in tumor diseases, GSDMD exhibits bidirectional regulatory functions, either suppressing tumor cell pyroptosis to promote tumor progression or modulating immune responses to inhibit tumor growth.

### GSDMD function in non-tumor diseases

GSDMD is broadly expressed and plays a pivotal regulatory role in non-tumor diseases, which can be categorized into infectious, non-infectious, and autoimmune diseases. Its function and mechanisms are closely associated with its localization in specific tissues and cell types. In non-infectious diseases, GSDMD activation is primarily driven by DAMPs released from injured tissues, dynamically influencing parenchymal cells and circulating or resident immune cells to induce inflammatory tissue damage. In infectious and autoimmune diseases, despite variations in activating factors, GSDMD predominantly regulates immune cell activity, resulting in inflammatory dysregulation and promoting disease progression.

#### Role of GSDMD in non-infectious diseases

GSDMD expression is significantly upregulated in heart, liver, kidney, nervous system, eye, and vascular diseases compared to normal controls. This upregulation mediates pyroptosis and triggers the release of inflammatory cytokines, leading to inflammatory damage and exacerbating disease progression.

##### Heart diseases

Ischemic heart disease, particularly MI/ R injury and MI, ranks among the most fatal cardiac conditions. Its pathological processes are closely linked to the expression and activation of GSDMD. In MI/ R injury, GSDMD plays a pivotal role in regulating cardiomyocyte pyroptosis, where its high expression exacerbates MI/ R damage. The underlying mechanism involves oxidative stress induced by MI/ R injury, which triggers the activation of caspase-11, leading to the cleavage of GSDMD. The cleaved GSDMD-N fragments then oligomerize to form membrane pores, contributing to cardiomyocyte damage [54]. In MI, neutrophils are recruited to the infarcted myocardium, where they undergo NETosis and release S100 Calcium Binding Protein A8 (S100A8) and S100 Calcium Binding Protein A9 (S100A9), upregulating C-X-C motif chemokine receptor 4 (CXCR4) expression in other neutrophils. This signaling drives neutrophils to migrate back to the bone marrow, where they accumulate and release IL-1β through GSDMD-mediated pyroptotic pores, thereby promoting granulopoiesis [61]. Moreover, GSDMD mediates IL-1β release via an autophagy-dependent mechanism, indicating that it may also have non-pyroptotic roles in MI pathophysiology [22].

In diabetic cardiomyopathy, upregulation of the mammalian target of rapamycin (mTOR) pathway accelerates pyroptosis, resulting in myocardial hypertrophy and collagen deposition, which further exacerbate cardiac dysfunction [63].

##### Liver disease

In liver diseases, the excessive activation of GSDMD significantly exacerbates liver injury, with its activation primarily involving immune cells and hepatocytes.

Among immune cells, invariant natural killer T (iNKT) cells, which reside in the liver, express high levels of caspase-1 and the tumor necrosis factor (TNF) superfamily receptor OX40. The activation of OX40 recruits the paracaspase MALT1 via TNF receptor-associated factor 6 (TRAF6), subsequently activating caspase-1. Activated caspase-1 cleaves GSDMD, promoting the maturation of IL-1β and the formation of pyroptotic pores. These pores release pro-inflammatory cytokines, thereby aggravating liver injury [64]. Additionally, infiltrating macrophages regulate inflammasome-mediated pyroptosis and hepatocyte damage through the Ikaros-SIRT1 axis, further intensifying liver injury in human liver transplant recipients and murine models of sterile hepatic inflammation [65].

Beyond immune cell-mediated liver injury, hepatocytes in patients with non-alcoholic fatty liver disease (NAFLD) and alcoholic steatohepatitis can directly activate the NLRP3-caspase-1 or DAG-PKCδ-NLRC4 inflammasomes, inducing pyroptosis. This process leads to the release of inflammasomes, which further promote liver fibrosis [25, 57–60]. Moreover, pyroptosis can also be initiated through the caspase-4/ 11 signaling pathway, activating downstream GSDMD. This mechanism exacerbates hepatocyte lytic death and drives polymorphonuclear (PMN) inflammation [55].

##### Kidney diseases

GSDMD promotes pyroptosis by mediating the activation of inflammasomes such as NLRP3, AIM2, and TLR4, thereby exacerbating kidney damage and impairing renal function.

APOL1 gene variants are significant risk factors for various forms of kidney dysfunction, and GSDMD has been identified as a key regulator in APOL1-associated kidney diseases. In these conditions, the activation of the NLRP3 inflammasome induces GSDMD-mediated pyroptosis, exacerbating proteinuria and accelerating renal function decline [70]. Similarly, in rhabdomyolysis-induced acute kidney injury (RIAKI), double-stranded DNA (dsDNA) released from damaged muscle tissue activates the AIM2 inflammasome signaling pathway, leading to macrophage pyroptosis. Notably, AIM2 deficiency results in macrophage accumulation, delayed renal recovery, and persistent kidney fibrosis. Conversely, upon dsDNA stimulation, macrophages expressing AIM2 rapidly undergo pyroptosis [178]. In diabetic kidney disease, tubular injury is associated with the upregulation of Toll-like receptor 4 (TLR4) and GSDMD. Pyroptosis in tubular cells is mediated through the activation of the TLR4/ NF-κB signaling pathway [179].

##### Nervous diseases

Neurological diseases can be broadly categorized into cerebrovascular diseases [26, 27, 68, 69], and neurodegenerative diseases [53, 62, 67], which are closely associated with the AIM2-caspase-1-GSDMD and NLRP3-caspase-1-GSDMD inflammasome signaling pathways.

In cerebrovascular diseases, AIM2-mediated pyroptosis is a key pathological feature. For instance, during ischemic stroke, brain ischemia leads to the release of double-stranded DNA (dsDNA) into the cytoplasm, activating the cyclic GMP-AMP synthase (cGAS) signaling pathway and the AIM2 inflammasome in microglia. This activation promotes the release of pro-inflammatory cytokines such as IL-1β, triggering neutrophil infiltration and ultimately resulting in neuronal death [26, 27, 69]. Additionally, analysis of cerebrospinal fluid (CSF) from patients with brain injury following subarachnoid hemorrhage (SAH) has revealed significantly elevated levels of AIM2 protein compared to controls, with these levels positively correlating with higher Hunt-Hess grades [68].

In contrast, neurodegenerative diseases are predominantly characterized by NLRP3-mediated pyroptosis. For example, in Alzheimer’s disease (AD), the loss of oligodendrocytes (OLs) and white matter degeneration are hallmark pathological features [180]. Mature OLs experience metabolic stress through the Drp1-HK1-NLRP3 signaling axis, leading to inflammation and OL damage, which causes demyelination, white matter degeneration, and cognitive impairment in AD models [67]. Similarly, activation of the NLRP3 inflammasome and subsequent GSDMD cleavage have been observed in the microglia of amyotrophic lateral sclerosis (ALS) patients and in Parkinson’s disease (PD) mouse models [53, 62].

##### Eye diseases

Pyroptosis mediated by GSDMD plays pathogenic roles in various eye diseases. In patients with cataracts, the expression levels of NLRP3, caspase-1, and GSDMD-N are significantly elevated in capsule tissues or cells. Downregulation of Cartilage acidic protein 1 (CRTAC1) has been shown to reduce ROS production and alleviate UVB-induced pyroptosis in human lens epithelial cells (HLECs) [181]. In age-related macular degeneration (AMD), degeneration of the retinal pigment epithelium (RPE) in macular lesions is driven by a caspase-4-mediated atypical inflammasome pathway. This mechanism involves the release of cytoplasmic mitochondrial DNA (mtDNA), which activates cyclic GMP-AMP synthase (cGAS) and subsequently triggers interferon-β (IFN-β) signaling [72]. In high myopia, axial elongation and refractive error progression are associated with elevated expression levels of NLRP3 and IL-1β [182, 183].

##### Vascular diseases

Atherosclerosis, characterized as an inflammatory disease associated with endothelial cell dysfunction, suppresses miR-223 expression through sequence complementarity with long non-coding RNA (lncRNA) MEG3. This suppression enhances NLRP3-GSDMD-mediated pyroptosis in endothelial cells, exacerbating the inflammatory damage linked to atherosclerosis [25]. Additionally, in Jak2VF macrophages, increased proliferation and glycolytic metabolism lead to DNA replication stress and activation of the AIM2 inflammasome, further contributing to vascular inflammation [24].

##### Others

The extracellular signal-related kinase (ERK)- NLRP3/ caspase-1 signaling pathway mediates pyroptosis in human nasal epithelial cells (hNECs) within the nasal mucosal tissue of patients with chronic rhinosinusitis, regardless of the presence of nasal polyps (CRSwNP). This pathway also contributes to glucocorticoid resistance in CRSwNP patients by disrupting the homeostasis of glucocorticoid receptors [73].

#### Role of GSDMD in infectious diseases

In human pathophysiology, the conflict between microbial infections and host immunity can lead to various diseases, many of which are associated with GSDMD-mediated pyroptosis [184].

##### Intestine diseases

GSDMD plays a dual role in colitis: it can exacerbate intestinal cell inflammatory damage, increasing pathological manifestations, or regulate immune cell-mediated inflammatory responses to reduce the occurrence of colitis.

In experimental colitis, GSDMD in colonic epithelial cells is activated via the caspase-8 inflammasome signaling pathway, facilitating the release of IL-1β through the non-lytic vesicle pathway [85]. Furthermore, caspase-8 and its ligand protein (Fas-associated death domain protein) FADD regulate Z-DNA binding protein 1 (ZBP1) and tumor necrosis factor receptor 1 (TNFR1)-mediated RIPK1 and RIPK3 signaling pathways in intestinal epithelial cells (IECs). FADD inhibits MLKL-induced necroptosis and caspase-8-GSDMD-dependent apoptosis-like epithelial cell death, thereby preventing IEC necrosis and subsequent intestinal inflammation [86]. In enteric neurons of inflammatory bowel disease, LPS enters the cytosol under the action of palmitic acid, activates caspase-11 and GSDMD, mediates pyroptosis and subsequent myoneural nitrergic neuron degeneration and colonic dysmotility [87].

In addition to enhancing pyroptosis, GSDMD in colonic macrophages regulates cGAS-mediated inflammatory responses, which protect against intestinal bacterial invasion and epithelial damage following mucosal barrier disruption, thus mitigating colitis [39].

##### Sepsis

Sepsis, a systemic inflammatory response syndrome caused by infection, is closely associated with pyroptosis mediated by the caspase-GSDMD pathway.

In cecal ligation and puncture (CLP)-induced septic mice, activation of the NLRP3 inflammasome triggers caspase-7 cleavage and pyroptosis, thereby mediating the inflammatory response [185]. In sepsis models induced by *Septicemia bacillus* α-toxin, the toxin binds to glycosylphosphatidylinositol (GPI)-anchored proteins on the host cell membrane, resulting in the release of magnesium and potassium ions. This ionic imbalance activates the NLRP3-caspase-1-GSDMD axis in macrophages, promoting the secretion of IL-1β and IL-18 [186]. In bacterial sepsis models treated with high doses of *Escherichia coli* lipopolysaccharide (LPS), pyroptosis is mediated through caspase-11 and caspase-8 activation [81]. Additionally, high-mobility group box 1 (HMGB1) interacts with LPS to mediate caspase-11-dependent pyroptosis [79]. Necroptosis mediated by RIPK3 and pyroptosis mediated by GSDMD work synergistically to amplify inflammatory signaling and tissue damage [80].

##### Others

In various infectious diseases, GSDMD-mediated pyroptosis amplifies inflammatory responses, in diverse cell types. This pyroptosis predominantly occurs in epithelial and immune cells.

In diabetic foot ulcers (DFUs) infected by *Staphylococcus aureus*, bacteria in the epidermis activate the AIM2- caspase-1 inflammasome, triggering pyroptosis. This process delays wound healing and prolongs inflammation in DFU patients [74]. During human rhinovirus (hRV) infection, inflammasome-mediated IL-1β secretion and pyroptosis in nasal epithelial progenitor cells and nasal epithelial cells depend on the DDX33/ DDX58- NLRP3- caspase-1- GSDMD axis. In differentiated human nasal epithelial cells (hNECs), hRV also stimulates the production of the major airway mucin MUC5AC through this pathway [77]. In endotoxemia, deletion of caspase-11 in endothelial cells significantly reduces LPS-induced pulmonary edema, neutrophil aggregation, and mortality [75].

In necrotizing fasciitis (NF) and streptococcal toxic shock syndrome (STSS) caused by *Group A Streptococcus* (GAS), the release of soluble M1 protein triggers macrophage (Mϕ) pyroptosis and IL-1β secretion through the NLRP3-caspase-1 pathway, leading to excessive inflammation and tissue damage [187]. In acute lung injury, flagellin induces IL-1β release by activating the NLRC4-caspase-1 inflammasome in alveolar macrophages. Inhibition of the NLRC4 inflammasome enhances the clearance of *Pseudomonas aeruginosa* [188]. In malaria, caspase-1/ 8 activity in monocytes promotes the release of TNF-α and IL-1β, key mediators of septic shock hypersensitivity and extracellular matrix development [76].

Beyond facilitating IL-1β release, GSDMD also mediates the release of IL-1α. For instance, in chronic *Toxoplasma* brain infections, microglia release the alarmin IL-1α via the GSDMD signaling pathway, promoting neuroinflammation and aiding in parasite control [82].

#### Role of GSDMD in autoimmune diseases

GSDMD plays a crucial role in the progression of several autoimmune diseases by mediating pyroptosis and amplifying inflammatory responses.

##### Multiple Sclerosis (MS)

The progression of experimental autoimmune encephalomyelitis (EAE), a widely used model for MS, depends on GSDMD activity [189]. MS is strongly associated with dysregulated inflammation mediated by the NLRP3- GSDMD pathway [89, 190]. In MS, particularly in the central nervous system (CNS) and peripheral vasculature, increased GSDMD expression is driven by the NLRP3 inflammasome pathway. This involves myeloid cells, such as macrophages and microglia, and oligodendrocytes (ODCs), where caspase-1-mediated inflammasome activation and pyroptosis exacerbate inflammatory demyelination [89]. Notably, GSDMD deficiency in myeloid cells reduces immune cell infiltration into the CNS, thereby mitigating neuroinflammation and demyelination [83]. In the EAE model, the NLRP3- caspase-1-GSDMD signaling pathway mediates the release of inflammatory cytokines, contributing to inflammation dysregulation [190].

##### Others

 Cryopyrin-Associated Periodic Syndromes (CAPS) are driven by missense mutations in the pyrin (NLRP3) gene, which result in hyperactive inflammasomes. This hyperactivation leads to the excessive production of pro-inflammatory cytokines, such as IL-1β and IL-18, driving disease pathology [191]. In familial Mediterranean fever (FMF), knock-in macrophages expressing chimeric Mefv (V726A) pyrin exhibit pyroptosis and GSDMD-mediated IL-1β secretion upon infection with *Clostridium difficile* [84, 192, 193]. Additionally, in a Kawasaki disease mouse model induced by Candida albicans cell wall extract (CAWS), NLRP3-dependent pyroptosis is activated in endothelial cells through the HMGB1/ RAGE/ cathepsin B signaling pathway [88].

### GSDMD function in tumor diseases

For tumor diseases, the role of GSDMD-mediated pyroptosis is highly complex, as it can both promote tumor growth and suppress tumorigenesis.

#### Lung cancer

In lung cancer, drugs induce pyroptosis by activating the caspase-1- GSDMD signaling pathway to inhibit tumor growth, though the specific mechanisms vary. For instance, in non-small cell lung cancer, Reniformin A enhances the stability of TLR4, upregulating its protein expression and activating the NLRP3- caspase-1- GSDMD pathway, thereby inducing pyroptosis in A549 cells and exerting anticancer effects [194]. Similarly, CuB binds to TLR4, promoting mitochondrial ROS generation, Tom20 accumulation, and calcium ion aggregation, which further facilitates pyroptosis [195]. Additionally, ZIF-8 nanoparticles induce pyroptosis in 4T1 cells via a caspase-1- GSDMD-dependent pathway, which activates antitumor immunity and reprograms the immunosuppressive tumor microenvironment, leading to effective tumor inhibition [196].

Notably, long non-coding RNAs (LncRNAs) play a critical role in tumor progression and therapeutic resistance. In gefitinib-resistant lung cancer cells, increased levels of H3K4me1 and H3K27Ac activate the expression of LINC00969. This LncRNA interacts with EZH2 and METTL3, regulating H3K27me3 modifications at the NLRP3 promoter at the transcriptional level and modulating m6A modifications of NLRP3 in an m6A- YTHDF2- dependent manner at the post-transcriptional level. These epigenetic modifications suppress NLRP3 expression, inhibit the activation of the NLRP3- caspase-1- GSDMD pathway, and confer an anti-pyroptotic phenotype to lung cancer cells [197].

#### Pancreatic cancer

In pancreatic cancer, GSDMD-mediated pyroptosis is a result of multi-organ interactions. Obesity, a significant risk factor for pancreatic cancer, induces macrophage pyroptosis through saturated fatty acids mediated by FABP4 in a caspase-1/ GSDMD-dependent manner. This process activates the NLRP3 inflammasome and IL-1β axis, further regulating epithelial-mesenchymal transition (EMT) signaling and promoting the migration, invasion, and metastasis of pancreatic cancer cells [198].

Dronedarone hydrochloride (DH) has been shown to increase mitochondrial stress in pancreatic cancer, leading to mitochondrial DNA (mtDNA) leakage and activation of the cGAS-STING pathway, thereby inducing pyroptosis in pancreatic cancer cells [199].

Furthermore, pancreatic cancer exhibits strong dependence on glucose and glutamine metabolism, and inhibition of their uptake results in nutrient deprivation and oxidative stress. This leads to a significant increase in ROS, activation of caspase-1 and GSDMD, and ultimately induces pyroptosis in pancreatic cancer cells [200].

#### Liver cancer

In hepatocellular carcinoma (HCC), positive GSDMD expression has been established as a marker of poor prognosis and identified as a critical driver of hepatocarcinogenesis. In HCC or metastatic liver tissues, HMGB1 binds to TLR4, activating the caspase-1/ GSDMD signaling pathway. GSDMD promotes autophagy via potassium efflux, thereby inhibiting cGAS activation, while calcium influx activates histone deacetylases and STAT1 signaling, inducing transcription of programmed death-ligand 1 (PD-L1) and enhancing its expression [201]. In mouse models of obesity-induced liver cancer, senescent hepatic stellate cells generate pores through caspase-11-mediated cleavage of GSDMD, allowing extracellular secretion of IL-1β and its family member IL-33. The secreted IL-33 further promotes hepatocarcinogenesis by activating ST2^+^ regulatory T cells (Tregs) [66].

#### Breast cancer

The endoplasmic reticulum stress sensor IRE1α is a critical regulator that suppresses the immunostimulatory effects of paclitaxel chemotherapy and inhibits innate immune recognition in immune-cold triple-negative breast cancer (TNBC). IRE1α RNase degrades paclitaxel-induced double-stranded RNA (dsRNA) via IRE1-dependent decay (RIDD), thereby preventing NLRP3 inflammasome-mediated pyroptosis. Inhibition of IRE1α allows paclitaxel to induce extensive dsRNA production, which is detected by ZBP1, leading to the activation of the NLRP3-GSDMD pathway and subsequent pyroptosis [202]. Furthermore, in TNBC patients, cisplatin (DDP) upregulates the long non-coding RNA maternal expressed gene 3 (lncRNA MEG3), which activates the NLRP3/ caspase-1/ GSDMD-mediated pyroptotic pathway [203].

#### Others

GSDMD can enhance tumor cell resistance to pyroptosis and augment the immune response of tumor cells.

For instance, GSDMD mediates tumor cell resistance in hypoxic environments. In complex cancer microenvironments, tumors construct hypoxic niches that upregulate ERRα expression. ERRα directly binds to the 3'-ACA ACT TGA ACA CGG AAA CG-5' sequence in the NLRP3 promoter, suppressing the caspase-1/ GSDMD signaling pathway and enhancing cellular resistance to pyroptosis [204]. Enhancer dysregulation is a recognized pro-tumorigenic mechanism, where the deletion of mixed-lineage leukemia 4 (MLL4) leads to the deactivation of enhancers and super-enhancers, reducing the expression of RNA-induced silencing complexes (RISC) and DNA methyltransferases. This reactivates transcriptional responses associated with double-stranded RNA (dsRNA)-interferon signaling and GSDMD-mediated pyroptosis [205].

GSDMD also mediates immunosuppression in the inflammatory tumor microenvironment (TIME). PARP inhibitors (PARPis) induce a specific type of pyroptosis in ovarian cancer cells via the TNF-caspase 8-GSDMD/E axis, enhancing TIME and promoting tumor-targeted immune responses [206]. In murine tumor models, DMB-induced low-level pyroptosis suppresses tumor growth without impairing the function of immune cells expressing GSDMD [207]. GSDMD could mediate pyroptosis in tumor cells. Intracellular polyamine depletion induces mitochondrial dysfunction, resulting in excessive mitochondrial copper ion accumulation and toxic protein aggregation, triggering tumor cell pyroptosis. Concurrently, mitochondrial reactive oxygen species (ROS) accumulation upregulates zDHHC5 and zDHHC9 expression, promoting the palmitoylation of GSDMD and its N-terminal fragment, thereby amplifying the pyroptotic response in tumor cells [208]. In colon cancer, GSDMD is activated in tumor cells through the ROS/ caspase-1 signaling pathway [209].

## Therapeutic targets and advances

GSDMD is extensively expressed in both non-tumor and tumor diseases, and its inhibition has shown considerable potential in alleviating disease pathology, positioning it as a promising therapeutic target. While several GSDMD-targeting inhibitors have been investigated in preclinical studies, none have advanced to clinical trials, underscoring the significant need and opportunity for further research and development in this field.

### GSDMD as a therapeutic target

GSDMD is a key regulator of inflammatory responses, widely expressed across various tissues and cell types with specific localization patterns. It plays a pivotal role in both non-tumor diseases and tumor-associated pathologies. In non-tumor diseases, GSDMD promotes pathogen clearance and tissue repair by regulating the release of inflammatory cytokines. While moderate inflammation facilitates tissue healing, excessive GSDMD expression during disease progression exacerbates hyperinflammatory responses and tissue damage. Studies using gene knockout models and pharmacological approaches demonstrate that inhibiting or knocking out GSDMD under pathological conditions significantly reduces pyroptosis and IL-1β release, effectively mitigating inflammation and tissue injury, without impairing physiological functions under normal conditions. These findings underscore the therapeutic potential of GSDMD as a target. However, in colitis, GSDMD in colonic macrophages mitigates epithelial damage caused by invasive intestinal pathogens via the cGAS signaling pathway, highlighting its protective role in immune defense [39]. This observation challenges the traditional view of GSDMD solely as a pro-inflammatory target.

In tumor diseases, GSDMD exhibits dual regulatory roles. On one hand, activating the GSDMD signaling pathway effectively suppresses tumor growth; on the other hand, pyroptosis may paradoxically promote tumor cell migration and metastasis. This dual functionality is primarily influenced by dynamic interactions among the tumor microenvironment, immune cells, and tumor cells [198].

In conclusion, GSDMD exerts broad effects in both tumor and non-tumor diseases, primarily through its regulation of inflammatory responses. Targeting GSDMD represents a promising strategy for mitigating inflammation-associated diseases. Current progress in this area is summarized in Table 2**.**

**Table 2:** Research progress of targeted inhibition of GSDMD in non-tumor diseases

Classic		Diseases	Validation of knockout mouse	Improvement	References
Non-infectious diseases	Heart diseases	Acute myocardial infarction	KO	Yes	[22, 61]
		Atherosclerosis	KO	Yes	[24, 25]
		Myocardial I/ R Injury	cKO (Cardiomyocyte knockout)	Yes	[54]
		Diabetic Cardiomyopathy	No	–	[63]
	Liver diseases	Acute and chronic liver diseases	KO	Yes	[64]
		Alcoholic hepatitis	No	–	[55]
		Alcoholic steatohepatitis	KO	Yes	[56]
		Liver warm ischemia–reperfusion injury	No	–	[65]
		Non-alcoholic steatohepatitis	KO	Yes	[57–59]
		Noninfectious liver injury	KO	Yes	[60]
		Obesity-associated hepatocellular carcinoma	No	Yes	[66]
	Nervous diseases	Alzheimer’s disease	No	–	[67]
		ALS	No	–	[53]
		Brain injury after subarachnoid haemorrhage	No	–	[68]
		Ischemic stroke	No	–	[26, 27]
		PD	No	–	[62]
		Vascular dementia	No	–	[69]
	Others	Chronic kidney diseases	KO/ inhibitors	Yes	[70]
		Acute kidney injury	No	–	[71]
		Age-related macular degeneration	KO	Yes	[72]
		CRSwNP	No	–	[73]
		Non-small cell lung cancer	No	–	[195]
Infectious diseases	Intestine diseases	Experimental colitis	KO	Yes	[85]
		Inflammatory bowel diseases	KO	Yes	[86]
		Western diet–induced colonic dysmotility	No	Yes	[87]
		Colitis	KO	No	[39]
	Others	DFUs	No	–	[74]
		Endotoxemia-induced lung injury	No	–	[75]
		Malaria	KO	Yes	[76]
		Rhinovirus infection	No	–	[77]
		Sepsis	KO	Yes	[78–80]
		Shock	KO	Yes	[81]
		Brain infections	KO	Yes	[82]
Autoimmune diseases		EAE	KO	Yes	[83]
		FMF	KO	Yes	[84]
		IBD	KO	Yes	[39, 85–87]
		Kawasaki disease	No	–	[88]
		Multiple sclerosis	siRNA	Yes	[89]

*ALS* Amyotrophic lateral sclerosis, *PD* Parkinson's disease, *CRSwNP* Chronic rhinosinusitis with nasal polyps, *DFUs* Diabetic foot ulcers, *FMF* Familial Mediterranean Fever, *IBD* Inflammatory bowel diseases

### Recent research advances in GSDMD inhibitors

#### Small molecular inhibitors

GSDMD inhibitors have been extensively studied for their therapeutic potential in inflammation-related diseases, with their molecular targets and mechanisms of action well-characterized. Most GSDMD inhibitors primarily target the human GSDMD-Cys191 or mouse GSDMD-Cys192 site, effectively suppressing GSDMD expression and GSDMD-N aggregation to block pyroptosis. Structurally, the GSDMD-Cys191/ 192 site is located at the distal end of the membrane-spanning region, specifically at the β8 chain's initiation point within the GSDMD β7—β8 hairpin structure. This site is crucial for pyroptotic β-tube formation [210]. Binding of inhibitors to this site occurs through hydrogen and covalent bonds, thereby disrupting the formation of pyroptotic β-tubes [37]. Small-molecule inhibitors such as necrosulfonamide, disulfiram, and fumarate bind to the GSDMD-Cys191/ 192 site, inhibiting GSDMD-N oligomerization and pyroptotic pore formation. These actions effectively block pyroptotic signaling pathways, reduce sepsis-related mortality, and alleviate tissue damage in EAE and MS models [22, 211–213]. Similarly, Danhong injection has been shown to suppress pyroptosis and mitigate myocardial fibrosis in MI by targeting this site with its active ingredient salvianolic acid E [214]. Furthermore, a selective GSDMD agonist, DMB, directly modifies Cys191, promoting GSDMD pore formation and pyroptosis without requiring GSDMD cleavage. This mechanism induces robust anti-tumor immunity with minimal toxicity [207].

Beyond direct binding to the Cys191/ 192 site, other inhibitors like LDC7559 and drug-free tea polyphenol nanoparticles (TPNs) target GSDMD-N activity and oligomerization. These inhibitors improve survival rates, mitigate hypothermia, and protect organ function in sepsis [146, 215]. The polyphenol punicalagin, derived from pomegranate, disrupts membrane fluidity and inhibits the insertion of GSDMD-NT [216].

**Fig. 5:**
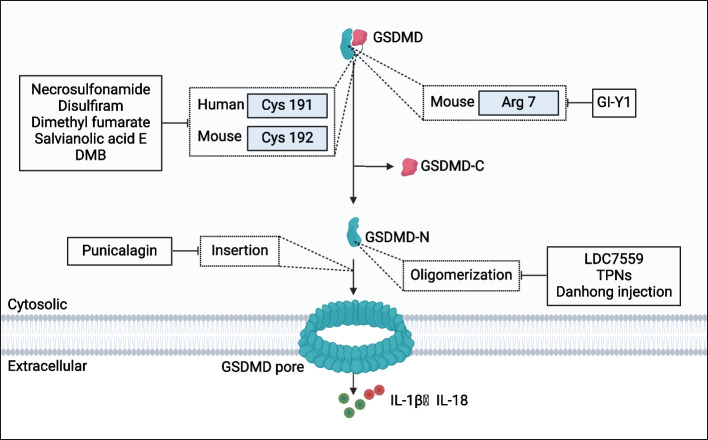
Summary of GSDMD Inhibitors. Necrosulfonamide, disulfonamide, dimethyl fumarate, salvianolic acid E, and DMB target the GSDMD Cys 191/192 site to suppress pyroptosis. GI-Y1 specifically targets GSDMD Arg 7 to inhibit pyroptosis. Punicalagin prevents the insertion of GSDMD-N into the membrane, disrupting pyroptosis initiation. LDC7559, TPNs, and Danhong injection inhibit pyroptosis by blocking GSDMD oligomerization

A novel target, GSDMD-Arg7, has also been identified for GSDMD inhibition. The inhibitor GI-Y1 prevents GSDMD-N aggregation on the membrane by binding to this site, thereby blocking pyroptotic pore formation and demonstrating therapeutic potential for cardiac diseases [217] **(**Fig. 5**)**.

Despite significant progress in the development of GSDMD inhibitors, no clinical trials have been registered on platforms such as ClinicalTrials.gov, and none of these inhibitors have received market approval. This gap underscores the need for further research and development, highlighting the substantial potential of GSDMD-targeting drugs for therapeutic applications.

#### Drug delivery formulations

Current research on GSDMD-based formulations mainly focuses on using nanomedicine to inhibit the GSDMD signaling pathway, thereby improving drug bioavailability and reducing adverse effects [218]. For instance, TPNs have been shown to alleviate sepsis-induced damage by inhibiting GSDMD oligomerization [215]. However, most GSDMD nanomedicines do not act by directly targeting the GSDMD protein but rather focus on inhibiting it ‘s signaling pathway. For example, Su et al. developed a carbonic anhydrase IX (CAIX)-anchored rhenium(I) photosensitizer (CA-Re) and found that it not only enabled type I and type II photodynamic therapy (PDT) under hypoxic conditions but also triggered GSDMD-mediated pyroptosis to enhance tumor immunogenicity [219]. Li et al. developed phospholipid-coated sodium citrate nanoparticles (PSCT NPs), which dissolve inside tumor cells, releasing large amounts of citrate and Na^+^ that activate both the caspase-1-GSDMD and caspase-8- Gasdemin C (GSDMC) pathways. These synergistic pathways induce robust pyroptosis, eliciting a significant antitumor immune response and inhibiting tumor growth [220]. Yang et al. designed tumor-targeting nanoparticles (CS-HAP@ATO NPs) by loading atorvastatin (ATO) onto chondroitin sulfate-modified hydroxyapatite nanoparticles (CS-HAP), which mediate inflammatory damage through the NLRP3- caspase-1- GSDMD pathway [221]. Similarly, ZIF-8 nanoparticles induce pyroptosis through a caspase-1- GSDMD-dependent pathway, triggering a tumor immune response and reprogramming the tumor's immunosuppressive microenvironment (TME) to effectively inhibit tumor growth [196]. Song et al. discovered that 2D NiCoOx nanosheets enhance pyroptosis and anticancer activity by inducing the ROS-NLRP3-GSDMD pathway [222]. Zhou et al. developed PG@Cu nanoparticles that reduce NOD-like receptor activation by inhibiting GSDMD oligomerization and NLRP3 activation [223]. Additionally, silver (Ag)-based antimicrobial nanoparticles stimulate microbial DNA leakage in the lungs, recruit caspase-1, promote pro-inflammatory cytokine release, and activate GSDMD, leading to severe pulmonary inflammation [224].

Moreover, a biomimetic nanoparticle platform (PDA@M) composed of a polydopamine core and macrophage membrane has been developed to inhibit NLRP3- caspase-1 signaling, thereby reducing pyroptosis and protecting the myocardium from oxidative stress induced by MI/ R [225]. Beyond nanomedicine, extracellular vesicles (EVs) have shown potential in delivering bioactive molecules that alter the phenotype of recipient cells. Genetically engineered mesenchymal stem cells (MSCs) were used to construct cavin-2-modified EVs, which exhibited increased uptake in TNF-α-treated nucleus pulposus cells (NPCs). In a three-dimensional hydrogel culture model, these modified EVs effectively ameliorated NPC pyroptosis, delaying the progression of intervertebral disc degeneration (IDD) [226].

While studies indicate that drug delivery via nanocarriers or targeted delivery systems can significantly enhance drug efficacy, current GSDMD nanomedicines remain functionally limited and fail to adequately account for the spatiotemporal dynamics of GSDMD in disease. Future formulation strategies may include ultrasound-assisted therapy, near-infrared light and magnetic field-controlled nanomedicine release platforms, and metal ion and pH-responsive systems to develop sustained-release and controlled-release formulations for GSDMD. Additionally, the application of Chimeric Antigen Receptor T-Cell Immunotherapy (CAR-T) technology offers new possibilities for the development of GSDMD inhibitors [218, 227–229].

## Conclusion and outlook

GSDMD exhibits significant tissue and cellular heterogeneity in its expression, function, and mechanisms. Under steady-state conditions, GSDMD is broadly expressed across various tissues and cell types, demonstrating specific spatial distribution and subtype specificity. In non-tumor diseases, elevated GSDMD expression exacerbates tissue inflammation. In tumor diseases, its expression patterns are more complex, potentially influenced by the tumor microenvironment. However, the transcriptional mechanisms driving these changes remain unclear.

GSDMD demonstrates diverse functions across diseases. In non-tumor diseases, GSDMD mediates inflammatory responses by releasing pro-inflammatory cytokines, aggravating tissue damage. However, in conditions such as colitis, GSDMD also plays a protective role by preventing epithelial damage and colitis onset [39]. In tumor diseases, GSDMD exhibits contradictory functions, potentially promoting or suppressing tumor progression depending on the context. Despite these complexities, GSDMD holds significant promise as a therapeutic target for inflammation-related diseases and cancers, with the development of GSDMD inhibitors presenting a wide range of applications.

To systematically uncover the therapeutic potential of GSDMD, this review examines the mechanisms underlying GSDMD-mediated diseases from the perspectives of structural biology, cell biology, and molecular biology. Structural studies have resolved the crystal structures of full-length GSDMD, GSDMD-N, GSDMD-C, GSDMD pores, and the GSDMD-caspase complex [31, 98, 230]. However, these static conformations do not account for potential conformational changes of GSDMD under pathological conditions. Moreover, GSDMD's expression and mechanisms differ among cell types, and whether its spatial conformation is influenced by cellular heterogeneity remains uncertain. Further structural investigations could elucidate its mechanisms and provide a theoretical basis for developing specific inhibitors.

The cellular and molecular mechanisms of GSDMD primarily involve its activation and cleavage, aggregation on cell membranes, pore formation, the release of pro-inflammatory cytokines, and the induction of pyroptosis. However, the GSDMD signaling pathway varies significantly across pathological microenvironments and cell types, characterized by: (1) Diversity in cleavage sites—In human macrophages, caspases-1, 4, 5, and 11 cleave GSDMD at Asp275 (272FLTD275), whereas in neutrophils, Cathepsin G cleaves it at Cys268 [1, 2, 32, 36, 37, 96, 148, 149]. (2) Mechanistic complexity—In MI, neutrophil GSDMD does not form pores to mediate IL-1β release but instead induces IL-1β release via autophagy, challenging the classical pyroptosis concept [38].

Despite progress in understanding GSDMD mechanisms, key limitations remain: (1) Insufficient focus on subcellular levels—In sepsis and atherosclerosis, macrophages release pro-inflammatory cytokines via the caspase-1- GSDMD pathway, triggering pyroptosis [1]. However, functional differences among macrophage subtypes (e.g., M1 vs. M2) in GSDMD-mediated mechanisms remain unexplored [231]. (2) Lack of dynamic studies—Although GSDMD expression shows temporal and spatial dependence during disease progression, systematic exploration of its dynamic changes in specific diseases is lacking. For example, in MI, the interplay between caspase-1 and caspase-11 pathways remains unresolved [232]. (3) Incomplete mechanistic understanding—In ischemia–reperfusion injury, GSDMD expression via the caspase-11 pathway mediates cardiomyocyte pyroptosis, but the specific triggers activating caspase-11 in cardiomyocytes are unknown [2].

Currently, several GSDMD inhibitors are in preclinical studies, but none have entered clinical trials. This underscores the importance of further research into GSDMD inhibitors, with significant potential for therapeutic development. Future therapeutic strategies should carefully consider factors such as therapeutic windows, selectivity, and safety.

In conclusion, as our understanding of GSDMD's expression, functions, and mechanisms deepens, and its potential as a therapeutic target becomes clearer, novel drugs and therapies targeting GSDMD could revolutionize treatment guidelines for related diseases.

## Data Availability

Not applicable. All figures are original.

## References

[1] Shi J, Zhao Y, Wang K, Shi X, Wang Y, Huang H, et al. Cleavage of GSDMD by inflammatory caspases determines pyroptotic cell death. Nature. 2015;526(7575):660–5. 10.1038/nature15514. 26375003 10.1038/nature15514

[2] Kayagaki N, Stowe IB, Lee BL, O’Rourke K, Anderson K, Warming S, et al. Caspase-11 cleaves gasdermin D for non-canonical inflammasome signalling. Nature. 2015;526(7575):666–71. 10.1038/nature15541. 26375259 10.1038/nature15541

[3] Shi P, Tang A, Xian L, Hou S, Zou D, Lv Y, et al. Loss of conserved Gsdma3 self-regulation causes autophagy and cell death. Biochem J. 2015;468(2):325–36. 10.1042/BJ20150204. 25825937 10.1042/BJ20150204

[4] Das S, Miller M, Beppu AK, Mueller J, McGeough MD, Vuong C, et al. GSDMB induces an asthma phenotype characterized by increased airway responsiveness and remodeling without lung inflammation. Proc Natl Acad Sci U S A. 2016;113(46):13132–7. 27799535 10.1073/pnas.1610433113 PMC5135378

[5] Saeki N, Usui T, Aoyagi K, Kim DH, Sato M, Mabuchi T, et al. Distinctive expression and function of four GSDM family genes (GSDMA-D) in normal and malignant upper gastrointestinal epithelium. Genes Chromosomes Cancer. 2009;48(3):261–71. 10.1002/gcc.20636. 19051310 10.1002/gcc.20636

[6] Hu Y, Jin S, Cheng L, Liu G, Jiang Q. Autoimmune disease variants regulate gene expression in human immune cells and whole blood. Proc Natl Acad Sci U S A. 2017;114(38):E7860–2. 10.1073/pnas.1712127114. 28882878 10.1073/pnas.1712127114 PMC5617311

[7] Watabe K, Ito A, Asada H, Endo Y, Kobayashi T, Nakamoto K, et al. Structure, expression and chromosome mapping of MLZE, a novel gene which is preferentially expressed in metastatic melanoma cells. Jpn J Cancer Res. 2001;92(2):140–51. 11223543 10.1111/j.1349-7006.2001.tb01076.x PMC5926699

[8] Tamura M, Tanaka S, Fujii T, Aoki A, Komiyama H, Ezawa K, et al. Members of a novel gene family, Gsdm, are expressed exclusively in the epithelium of the skin and gastrointestinal tract in a highly tissue-specific manner. Genomics. 2007;89(5):618–29. 17350798 10.1016/j.ygeno.2007.01.003

[9] Miguchi M, Hinoi T, Shimomura M, Adachi T, Saito Y, Niitsu H, et al. Gasdermin C Is Upregulated by Inactivation of Transforming Growth Factor β Receptor Type II in the Presence of Mutated Apc, Promoting Colorectal Cancer Proliferation. PLoS ONE. 2016;11(11): e0166422. 10.1371/journal.pone.0166422. 27835699 10.1371/journal.pone.0166422 PMC5105946

[10] Zhang Z and Lieberman J. Lighting a fire on the reef*.* Science Immunology. 2020;5(54) 10.1126/sciimmunol.abf0905. 10.1126/sciimmunol.abf0905 PMC7900804 33277372

[11] Jiang S, Zhou Z, Sun Y, Zhang T, and Sun L. Coral gasdermin triggers pyroptosis*.* Science Immunology. 2020;5(54) 10.1126/sciimmunol.abd2591. 10.1126/sciimmunol.abd2591 33277371

[12] Galluzzi L, Vitale I, Aaronson SA, Abrams JM, Adam D, Agostinis P, et al. Molecular mechanisms of cell death: recommendations of the Nomenclature Committee on Cell Death 2018. Cell Death Differ. 2018;25(3):486–541. 10.1038/s41418-017-0012-4. 29362479 10.1038/s41418-017-0012-4 PMC5864239

[13] Friedlander AM. Macrophages are sensitive to anthrax lethal toxin through an acid-dependent process. J Biol Chem. 1986;261(16):7123–6. 3711080

[14] Black RA, Kronheim SR, Merriam JE, March CJ, Hopp TP. A pre-aspartate-specific protease from human leukocytes that cleaves pro-interleukin-1 beta. J Biol Chem. 1989;264(10):5323–6. 2784432

[15] Cookson BT, Brennan MA. Pro-inflammatory programmed cell death. Trends Microbiol. 2001;9(3):113–4. 11303500 10.1016/s0966-842x(00)01936-3

[16] Katoh M, Katoh M. Identification and characterization of human DFNA5L, mouse Dfna5l, and rat Dfna5l genes in silico. Int J Oncol. 2004;25(3):765–70. 15289881

[17] Ding J, Wang K, Liu W, She Y, Sun Q, Shi J, et al. Pore-forming activity and structural autoinhibition of the gasdermin family. Nature. 2016;535(7610):111–6. 10.1038/nature18590. 27281216 10.1038/nature18590

[18] Liu X, Zhang Z, Ruan J, Pan Y, Magupalli VG, Wu H, et al. Inflammasome-activated gasdermin D causes pyroptosis by forming membrane pores. Nature. 2016;535(7610):153–8. 10.1038/nature18629. 27383986 10.1038/nature18629 PMC5539988

[19] Sborgi L, Rühl S, Mulvihill E, Pipercevic J, Heilig R, Stahlberg H, et al. GSDMD membrane pore formation constitutes the mechanism of pyroptotic cell death*.* The EMBO Journal. 2016;35(16):1766–78. 10.15252/embj.201694696. 10.15252/embj.201694696 PMC5010048 27418190

[20] Aglietti RA, Estevez A, Gupta A, Ramirez MG, Liu PS, Kayagaki N, et al. GsdmD p30 elicited by caspase-11 during pyroptosis forms pores in membranes. Proc Natl Acad Sci U S A. 2016;113(28):7858–63. 10.1073/pnas.1607769113. 27339137 10.1073/pnas.1607769113 PMC4948338

[21] Ruan J, Xia S, Liu X, Lieberman J, Wu H. Cryo-EM structure of the gasdermin A3 membrane pore. Nature. 2018;557(7703):62–7. 10.1038/s41586-018-0058-6. 29695864 10.1038/s41586-018-0058-6 PMC6007975

[22] Jiang K, Tu Z, Chen K, Xu Y, Chen F, Xu S, et al. Gasdermin D inhibition confers antineutrophil-mediated cardioprotection in acute myocardial infarction*.* J Clin Invest. 2022;132(1) 10.1172/JCI151268. 10.1172/JCI151268 PMC8718151 34752417

[23] Aliaga J, Bonaventura A, Mezzaroma E, Dhakal Y, Mauro A G, Abbate A, et al. Preservation of Contractile Reserve and Diastolic Function by Inhibiting the NLRP3 Inflammasome with OLT1177® (Dapansutrile) in a Mouse Model of Severe Ischemic Cardiomyopathy Due to Non-Reperfused Anterior Wall Myocardial Infarction*.* Molecules. 2021;26(12) 10.3390/molecules26123534. 10.3390/molecules26123534 PMC8227554 34207886

[24] Fidler TP, Xue C, Yalcinkaya M, Hardaway B, Abramowicz S, Xiao T, et al. The AIM2 inflammasome exacerbates atherosclerosis in clonal haematopoiesis. Nature. 2021;592(7853):296–301. 10.1038/s41586-021-03341-5. 33731931 10.1038/s41586-021-03341-5 PMC8038646

[25] Zhang Y, Liu X, Bai X, Lin Y, Li Z, Fu J, et al. Melatonin prevents endothelial cell pyroptosis via regulation of long noncoding RNA MEG3/miR-223/NLRP3 axis*.* Journal of Pineal Research. 2018;64(2) 10.1111/jpi.12449. 10.1111/jpi.12449 29024030

[26] Li Q, Cao Y, Dang C, Han B, Han R, Ma H, et al. Inhibition of double-strand DNA-sensing cGAS ameliorates brain injury after ischemic stroke*.* EMBO Molecular Medicine. 2020;12(4):e11002. 10.15252/emmm.201911002. 10.15252/emmm.201911002 PMC7136961 32239625

[27] Kim H, Kim M J, Kwon Y W, Jeon S, Lee S-Y, Kim C-S, et al. Benefits of a Skull-Interfaced Flexible and Implantable Multilight Emitting Diode Array for Photobiomodulation in Ischemic Stroke*.* Advanced Science (Weinheim, Baden-Wurttemberg, Germany). 2022;9(11):e2104629. 10.1002/advs.202104629. 10.1002/advs.202104629 PMC9008794 35076161

[28] Liu X, Xia S, Zhang Z, Wu H, Lieberman J. Channelling inflammation: gasdermins in physiology and disease. Nat Rev Drug Discovery. 2021;20(5):384–405. 10.1038/s41573-021-00154-z. 33692549 10.1038/s41573-021-00154-z PMC7944254

[29] Uhlen M, Zhang C, Lee S, Sjöstedt E, Fagerberg L, Bidkhori G, et al. A pathology atlas of the human cancer transcriptome*.* Science (New York, N.Y.). 2017;357(6352) 10.1126/science.aan2507. 10.1126/science.aan2507 28818916

[30] Kuang S, Zheng J, Yang H, Li S, Duan S, Shen Y, et al. Structure insight of GSDMD reveals the basis of GSDMD autoinhibition in cell pyroptosis. Proc Natl Acad Sci U S A. 2017;114(40):10642–7. 10.1073/pnas.1708194114. 28928145 10.1073/pnas.1708194114 PMC5635896

[31] Liu Z, Wang C, Yang J, Zhou B, Yang R, Ramachandran R, et al. Crystal Structures of the Full-Length Murine and Human Gasdermin D Reveal Mechanisms of Autoinhibition, Lipid Binding, and Oligomerization*.* Immunity. 2019;51(1) 10.1016/j.immuni.2019.04.017. 10.1016/j.immuni.2019.04.017 PMC6640092 31097341

[32] He W-t, Wan H, Hu L, Chen P, Wang X, Huang Z, et al. Gasdermin D is an executor of pyroptosis and required for interleukin-1β secretion*.* Cell Research. 2015;25(12):1285–98. 10.1038/cr.2015.139. 10.1038/cr.2015.139 PMC4670995 26611636

[33] Orning P, Weng D, Starheim K, Ratner D, Best Z, Lee B, et al. Pathogen blockade of TAK1 triggers caspase-8-dependent cleavage of gasdermin D and cell death*.* Science (New York, N.Y.). 2018;362(6418):1064–69. 10.1126/science.aau2818. 10.1126/science.aau2818 PMC6522129 30361383

[34] Sarhan J, Liu BC, Muendlein HI, Li P, Nilson R, Tang AY, et al. Caspase-8 induces cleavage of gasdermin D to elicit pyroptosis during Yersinia infection. Proc Natl Acad Sci U S A. 2018;115(46):E10888–97. 10.1073/pnas.1809548115. 30381458 10.1073/pnas.1809548115 PMC6243247

[35] Zheng Z, Deng W, Bai Y, Miao R, Mei S, Zhang Z, et al. The Lysosomal Rag-Ragulator Complex Licenses RIPK1 and Caspase-8-mediated Pyroptosis by Yersinia*.* Science (New York, N.Y.). 2021;372(6549) 10.1126/science.abg0269. 10.1126/science.abg0269 PMC8769499 35058659

[36] Liu Z, Wang C, Yang J, Chen Y, Zhou B, Abbott D W, et al. Caspase-1 Engages Full-Length Gasdermin D through Two Distinct Interfaces That Mediate Caspase Recruitment and Substrate Cleavage*.* Immunity. 2020;53(1) 10.1016/j.immuni.2020.06.007. 10.1016/j.immuni.2020.06.007 PMC7382298 32553275

[37] Kambara H, Liu F, Zhang X, Liu P, Bajrami B, Teng Y, et al. Gasdermin D Exerts Anti-inflammatory Effects by Promoting Neutrophil Death. Cell Rep. 2018;22(11):2924–36. 10.1016/j.celrep.2018.02.067. 29539421 10.1016/j.celrep.2018.02.067 PMC5878047

[38] Karmakar M, Minns M, Greenberg EN, Diaz-Aponte J, Pestonjamasp K, Johnson JL, et al. N-GSDMD trafficking to neutrophil organelles facilitates IL-1β release independently of plasma membrane pores and pyroptosis. Nat Commun. 2020;11(1):2212. 10.1038/s41467-020-16043-9. 32371889 10.1038/s41467-020-16043-9 PMC7200749

[39] Ma C, Yang D, Wang B, Wu C, Wu Y, Li S, et al. Gasdermin D in macrophages restrains colitis by controlling cGAS-mediated inflammation*.* Science Advances. 2020;6(21):eaaz6717. 10.1126/sciadv.aaz6717. 10.1126/sciadv.aaz6717 PMC7314554 32671214

[40] Lake BB, Chen S, Hoshi M, Plongthongkum N, Salamon D, Knoten A, et al. A single-nucleus RNA-sequencing pipeline to decipher the molecular anatomy and pathophysiology of human kidneys. Nat Commun. 2019;10(1):2832. 10.1038/s41467-019-10861-2. 31249312 10.1038/s41467-019-10861-2 PMC6597610

[41] Elmentaite R, Kumasaka N, Roberts K, Fleming A, Dann E, King HW, et al. Cells of the human intestinal tract mapped across space and time. Nature. 2021;597(7875):250–5. 10.1038/s41586-021-03852-1. 34497389 10.1038/s41586-021-03852-1 PMC8426186

[42] MacParland SA, Liu JC, Ma X-Z, Innes BT, Bartczak AM, Gage BK, et al. Single cell RNA sequencing of human liver reveals distinct intrahepatic macrophage populations. Nat Commun. 2018;9(1):4383. 10.1038/s41467-018-06318-7. 30348985 10.1038/s41467-018-06318-7 PMC6197289

[43] Ben-Moshe S, Itzkovitz S. Spatial heterogeneity in the mammalian liver. Nat Rev Gastroenterol Hepatol. 2019;16(7):395–410. 10.1038/s41575-019-0134-x. 30936469 10.1038/s41575-019-0134-x

[44] Liu X, Lieberman J. Knocking ’em Dead: Pore-Forming Proteins in Immune Defense. Annu Rev Immunol. 2020;38:455–85. 10.1146/annurev-immunol-111319-023800. 32004099 10.1146/annurev-immunol-111319-023800 PMC7260445

[45] Shi J, Zhao Y, Wang Y, Gao W, Ding J, Li P, et al. Inflammatory caspases are innate immune receptors for intracellular LPS. Nature. 2014;514(7521):187–92. 10.1038/nature13683. 25119034 10.1038/nature13683

[46] Han X, Wang R, Zhou Y, Fei L, Sun H, Lai S, et al. Mapping the Mouse Cell Atlas by Microwell-Seq*.* Cell. 2018;172(5) 10.1016/j.cell.2018.02.001. 10.1016/j.cell.2018.02.001 29474909

[47] De Schutter E, Roelandt R, Riquet FB, Van Camp G, Wullaert A, Vandenabeele P. Punching Holes in Cellular Membranes: Biology and Evolution of Gasdermins. Trends Cell Biol. 2021;31(6):500–13. 10.1016/j.tcb.2021.03.004. 33771452 10.1016/j.tcb.2021.03.004

[48] Pinto AR, Ilinykh A, Ivey MJ, Kuwabara JT, D’Antoni ML, Debuque R, et al. Revisiting Cardiac Cellular Composition. Circ Res. 2016;118(3):400–9. 10.1161/CIRCRESAHA.115.307778. 26635390 10.1161/CIRCRESAHA.115.307778 PMC4744092

[49] Oetjen K A, Lindblad K E, Goswami M, Gui G, Dagur P K, Lai C, et al. Human bone marrow assessment by single-cell RNA sequencing, mass cytometry, and flow cytometry*.* JCI Insight. 2018;3(23) 10.1172/jci.insight.124928. 10.1172/jci.insight.124928 PMC6328018 30518681

[50] Liao J, Yu Z, Chen Y, Bao M, Zou C, Zhang H, et al. Single-cell RNA sequencing of human kidney. Sci Data. 2020;7(1):4. 10.1038/s41597-019-0351-8. 31896769 10.1038/s41597-019-0351-8 PMC6940381

[51] Travaglini KJ, Nabhan AN, Penland L, Sinha R, Gillich A, Sit RV, et al. A molecular cell atlas of the human lung from single-cell RNA sequencing. Nature. 2020;587(7835):619–25. 10.1038/s41586-020-2922-4. 33208946 10.1038/s41586-020-2922-4 PMC7704697

[52] Yap J, Irei J, Lozano-Gerona J, Vanapruks S, Bishop T, Boisvert WA. Macrophages in cardiac remodelling after myocardial infarction. Nat Rev Cardiol. 2023;20(6):373–85. 10.1038/s41569-022-00823-5. 36627513 10.1038/s41569-022-00823-5

[53] Van Schoor E, Ospitalieri S, Moonen S, Tomé SO, Ronisz A, Ok O, et al. Increased pyroptosis activation in white matter microglia is associated with neuronal loss in ALS motor cortex. Acta Neuropathol. 2022;144(3):393–411. 10.1007/s00401-022-02466-9. 35867112 10.1007/s00401-022-02466-9

[54] Shi H, Gao Y, Dong Z, Yang J e, Gao R, Li X, et al. GSDMD-Mediated Cardiomyocyte Pyroptosis Promotes Myocardial I/R Injury*.* Circ Res. 2021;129(3):383–96. 10.1161/CIRCRESAHA.120.318629. 10.1161/CIRCRESAHA.120.318629 PMC8291144 34015941

[55] Khanova E, Wu R, Wang W, Yan R, Chen Y, French S W, et al. Pyroptosis by caspase11/4-gasdermin-D pathway in alcoholic hepatitis in mice and patients*.* Hepatology (Baltimore, Md.). 2018;67(5):1737–53. 10.1002/hep.29645. 10.1002/hep.29645 PMC5906140 29108122

[56] Luan J, Chen W, Fan J, Wang S, Zhang X, Zai W, et al. GSDMD membrane pore is critical for IL-1β release and antagonizing IL-1β by hepatocyte-specific nanobiologics is a promising therapeutics for murine alcoholic steatohepatitis. Biomaterials. 2020;227: 119570. 10.1016/j.biomaterials.2019.119570. 31670032 10.1016/j.biomaterials.2019.119570

[57] Gaul S, Leszczynska A, Alegre F, Kaufmann B, Johnson CD, Adams LA, et al. Hepatocyte pyroptosis and release of inflammasome particles induce stellate cell activation and liver fibrosis. J Hepatol. 2021;74(1):156–67. 10.1016/j.jhep.2020.07.041. 32763266 10.1016/j.jhep.2020.07.041 PMC7749849

[58] Xu B, Jiang M, Chu Y, Wang W, Chen D, Li X, et al. Gasdermin D plays a key role as a pyroptosis executor of non-alcoholic steatohepatitis in humans and mice. J Hepatol. 2018;68(4):773–82. 10.1016/j.jhep.2017.11.040. 29273476 10.1016/j.jhep.2017.11.040

[59] Koh EH, Yoon JE, Ko MS, Leem J, Yun J-Y, Hong CH, et al. Sphingomyelin synthase 1 mediates hepatocyte pyroptosis to trigger non-alcoholic steatohepatitis. Gut. 2021;70(10):1954–64. 10.1136/gutjnl-2020-322509. 33208407 10.1136/gutjnl-2020-322509 PMC8458090

[60] Yang C, Sun P, Deng M, Loughran P, Li W, Yi Z, et al. Gasdermin D protects against noninfectious liver injury by regulating apoptosis and necroptosis. Cell Death Dis. 2019;10(7):481. 10.1038/s41419-019-1719-6. 31209224 10.1038/s41419-019-1719-6 PMC6579760

[61] Sreejit G, Nooti SK, Jaggers RM, Athmanathan B, Ho Park K, Al-Sharea A, et al. Retention of the NLRP3 Inflammasome-Primed Neutrophils in the Bone Marrow Is Essential for Myocardial Infarction-Induced Granulopoiesis. Circulation. 2022;145(1):31–44. 10.1161/CIRCULATIONAHA.121.056019. 34788059 10.1161/CIRCULATIONAHA.121.056019 PMC8716427

[62] Ma X, Hao J, Wu J, Li Y, Cai X, and Zheng Y. Prussian Blue Nanozyme as a Pyroptosis Inhibitor Alleviates Neurodegeneration*.* Advanced Materials (Deerfield Beach, Fla.). 2022;34(15):e2106723. 10.1002/adma.202106723. 10.1002/adma.202106723 35143076

[63] Yang F, Qin Y, Wang Y, Meng S, Xian H, Che H, et al. Metformin Inhibits the NLRP3 Inflammasome via AMPK/mTOR-dependent Effects in Diabetic Cardiomyopathy. Int J Biol Sci. 2019;15(5):1010–9. 10.7150/ijbs.29680. 31182921 10.7150/ijbs.29680 PMC6535781

[64] Lan P, Fan Y, Zhao Y, Lou X, Monsour HP, Zhang X, et al. TNF superfamily receptor OX40 triggers invariant NKT cell pyroptosis and liver injury. J Clin Invest. 2017;127(6):2222–34. 10.1172/JCI91075. 28436935 10.1172/JCI91075 PMC5451219

[65] Kadono K, Kageyama S, Nakamura K, Hirao H, Ito T, Kojima H, et al. Myeloid Ikaros-SIRT1 signaling axis regulates hepatic inflammation and pyroptosis in ischemia-stressed mouse and human liver. J Hepatol. 2022;76(4):896–909. 10.1016/j.jhep.2021.11.026. 34871625 10.1016/j.jhep.2021.11.026 PMC9704689

[66] Yamagishi R, Kamachi F, Nakamura M, Yamazaki S, Kamiya T, Takasugi M, et al. Gasdermin D-mediated release of IL-33 from senescent hepatic stellate cells promotes obesity-associated hepatocellular carcinoma*.* Sci Immunol. 2022;7(72):eabl7209. 10.1126/sciimmunol.abl7209. 10.1126/sciimmunol.abl7209 35749514

[67] Zhang X, Wang R, Hu D, Sun X, Fujioka H, Lundberg K, et al. Oligodendroglial glycolytic stress triggers inflammasome activation and neuropathology in Alzheimer's disease*.* Science Advances. 2020;6(49) 10.1126/sciadv.abb8680. 10.1126/sciadv.abb8680 PMC7717916 33277246

[68] Yuan B, Zhou X-M, You Z-Q, Xu W-D, Fan J-M, Chen S-J, et al. Inhibition of AIM2 inflammasome activation alleviates GSDMD-induced pyroptosis in early brain injury after subarachnoid haemorrhage. Cell Death Dis. 2020;11(1):76. 10.1038/s41419-020-2248-z. 32001670 10.1038/s41419-020-2248-z PMC6992766

[69] Poh L, Fann DY, Wong P, Lim HM, Foo SL, Kang S-W, et al. AIM2 inflammasome mediates hallmark neuropathological alterations and cognitive impairment in a mouse model of vascular dementia. Mol Psychiatry. 2021;26(8):4544–60. 10.1038/s41380-020-00971-5. 33299135 10.1038/s41380-020-00971-5

[70] Wu J, Raman A, Coffey N J, Sheng X, Wahba J, Seasock M J, et al. The key role of NLRP3 and STING in APOL1-associated podocytopathy*.* J Clin Invest. 2021;131(20) 10.1172/JCI136329. 10.1172/JCI136329 PMC8516463 34651582

[71] Zhang Z, Shao X, Jiang N, Mou S, Gu L, Li S, et al. Caspase-11-mediated tubular epithelial pyroptosis underlies contrast-induced acute kidney injury. Cell Death Dis. 2018;9(10):983. 10.1038/s41419-018-1023-x. 30250284 10.1038/s41419-018-1023-x PMC6155357

[72] Kerur N, Fukuda S, Banerjee D, Kim Y, Fu D, Apicella I, et al. cGAS drives noncanonical-inflammasome activation in age-related macular degeneration. Nat Med. 2018;24(1):50–61. 10.1038/nm.4450. 29176737 10.1038/nm.4450 PMC5760363

[73] Li Y, Chang L-H, Huang W-Q, Bao H-W, Li X, Chen X-H, et al. IL-17A mediates pyroptosis via the ERK pathway and contributes to steroid resistance in CRSwNP. J Allergy Clin Immunol. 2022;150(2):337–51. 10.1016/j.jaci.2022.02.031. 35346673 10.1016/j.jaci.2022.02.031

[74] Pastar I, Sawaya A P, Marjanovic J, Burgess J L, Strbo N, Rivas K E, et al. Intracellular Staphylococcus aureus triggers pyroptosis and contributes to inhibition of healing due to perforin-2 suppression*.* J Clin Invest. 2021;131(24) 10.1172/JCI133727. 10.1172/JCI133727 PMC8670843 34730110

[75] Cheng KT, Xiong S, Ye Z, Hong Z, Di A, Tsang KM, et al. Caspase-11-mediated endothelial pyroptosis underlies endotoxemia-induced lung injury. J Clin Invest. 2017;127(11):4124–35. 10.1172/JCI94495. 28990935 10.1172/JCI94495 PMC5663346

[76] Pereira LMN, Assis PA, de Araújo NM, Durso DF, Junqueira C, Ataíde MA, et al. Caspase-8 mediates inflammation and disease in rodent malaria. Nat Commun. 2020;11(1):4596. 10.1038/s41467-020-18295-x. 32929083 10.1038/s41467-020-18295-x PMC7490701

[77] Liu T, Zhou Y T, Wang L Q, Li L Y, Bao Q, Tian S, et al. NOD-like receptor family, pyrin domain containing 3 (NLRP3) contributes to inflammation, pyroptosis, and mucin production in human airway epithelium on rhinovirus infection*.* The Journal of Allergy and Clinical Immunology. 2019;144(3) 10.1016/j.jaci.2019.05.006. 10.1016/j.jaci.2019.05.006 31102698

[78] Jing W, Pilato J L, Kay C, Feng S, Tuipulotu D E, Mathur A, et al. α-toxin activates the NLRP3 inflammasome by engaging GPI-anchored proteins*.* Science Immunology. 2022;7(71):eabm1803. 10.1126/sciimmunol.abm1803. 10.1126/sciimmunol.abm1803 35594341

[79] Deng M, Tang Y, Li W, Wang X, Zhang R, Zhang X, et al. The Endotoxin Delivery Protein HMGB1 Mediates Caspase-11-Dependent Lethality in Sepsis*.* Immunity. 2018;49(4) 10.1016/j.immuni.2018.08.016. 10.1016/j.immuni.2018.08.016 PMC6300139 30314759

[80] Chen H, Li Y, Wu J, Li G, Tao X, Lai K, et al. RIPK3 collaborates with GSDMD to drive tissue injury in lethal polymicrobial sepsis. Cell Death Differ. 2020;27(9):2568–85. 10.1038/s41418-020-0524-1. 32152555 10.1038/s41418-020-0524-1 PMC7429874

[81] Mandal P, Feng Y, Lyons J D, Berger S B, Otani S, DeLaney A, et al. Caspase-8 Collaborates with Caspase-11 to Drive Tissue Damage and Execution of Endotoxic Shock*.* Immunity. 2018;49(1) 10.1016/j.immuni.2018.06.011. 10.1016/j.immuni.2018.06.011 PMC6064639 30021146

[82] Batista S J, Still K M, Johanson D, Thompson J A, OʼBrien C A, Lukens J R, et al. Gasdermin-D-dependent IL-1α release from microglia promotes protective immunity during chronic Toxoplasma gondii infection*.* Nature Communications. 2020;11(1):3687. 10.1038/s41467-020-17491-z. 10.1038/s41467-020-17491-z PMC7378823 32703941

[83] Li S, Wu Y, Yang D, Wu C, Ma C, Liu X, et al. Gasdermin D in peripheral myeloid cells drives neuroinflammation in experimental autoimmune encephalomyelitis. J Exp Med. 2019;216(11):2562–81. 10.1084/jem.20190377. 31467036 10.1084/jem.20190377 PMC6829591

[84] Kanneganti A, Malireddi RKS, Saavedra PHV, Vande Walle L, Van Gorp H, Kambara H, et al. GSDMD is critical for autoinflammatory pathology in a mouse model of Familial Mediterranean Fever. J Exp Med. 2018;215(6):1519–29. 10.1084/jem.20172060. 29793924 10.1084/jem.20172060 PMC5987922

[85] Bulek K, Zhao J, Liao Y, Rana N, Corridoni D, Antanaviciute A, et al. Epithelial-derived gasdermin D mediates nonlytic IL-1β release during experimental colitis. J Clin Invest. 2020;130(8):4218–34. 10.1172/JCI138103. 32597834 10.1172/JCI138103 PMC7410065

[86] Schwarzer R, Jiao H, Wachsmuth L, Tresch A, and Pasparakis M. FADD and Caspase-8 Regulate Gut Homeostasis and Inflammation by Controlling MLKL- and GSDMD-Mediated Death of Intestinal Epithelial Cells*.* Immunity. 2020;52(6) 10.1016/j.immuni.2020.04.002. 10.1016/j.immuni.2020.04.002 32362323

[87] Ye L, Li G, Goebel A, Raju AV, Kong F, Lv Y, et al. Caspase-11-mediated enteric neuronal pyroptosis underlies Western diet-induced colonic dysmotility. J Clin Invest. 2020;130(7):3621–36. 10.1172/JCI130176. 32484462 10.1172/JCI130176 PMC7324173

[88] Jia C, Zhang J, Chen H, Zhuge Y, Chen H, Qian F, et al. Endothelial cell pyroptosis plays an important role in Kawasaki disease via HMGB1/RAGE/cathespin B signaling pathway and NLRP3 inflammasome activation. Cell Death Dis. 2019;10(10):778. 10.1038/s41419-019-2021-3. 31611559 10.1038/s41419-019-2021-3 PMC6791856

[89] McKenzie BA, Mamik MK, Saito LB, Boghozian R, Monaco MC, Major EO, et al. Caspase-1 inhibition prevents glial inflammasome activation and pyroptosis in models of multiple sclerosis. Proc Natl Acad Sci U S A. 2018;115(26):E6065–74. 10.1073/pnas.1722041115. 29895691 10.1073/pnas.1722041115 PMC6042136

[90] Hou J, Hsu J-M, Hung M-C. Molecular mechanisms and functions of pyroptosis in inflammation and antitumor immunity. Mol Cell. 2021;81(22):4579–90. 10.1016/j.molcel.2021.09.003. 34562371 10.1016/j.molcel.2021.09.003 PMC8604761

[91] Deng W, Bai Y, Deng F, Pan Y, Mei S, Zheng Z, et al. Streptococcal pyrogenic exotoxin B cleaves GSDMA and triggers pyroptosis. Nature. 2022;602(7897):496–502. 10.1038/s41586-021-04384-4. 35110732 10.1038/s41586-021-04384-4 PMC9703647

[92] LaRock DL, Johnson AF, Wilde S, Sands JS, Monteiro MP, LaRock CN. Group A Streptococcus induces GSDMA-dependent pyroptosis in keratinocytes. Nature. 2022;605(7910):527–31. 10.1038/s41586-022-04717-x. 35545676 10.1038/s41586-022-04717-x PMC9186297

[93] Zhou Z, He H, Wang K, Shi X, Wang Y, Su Y, et al. Granzyme A from cytotoxic lymphocytes cleaves GSDMB to trigger pyroptosis in target cells*.* Science. 2020;368(6494) 10.1126/science.aaz7548. 10.1126/science.aaz7548 32299851

[94] Hou J, Zhao R, Xia W, Chang C-W, You Y, Hsu J-M, et al. PD-L1-mediated gasdermin C expression switches apoptosis to pyroptosis in cancer cells and facilitates tumour necrosis. Nat Cell Biol. 2020;22(10):1264–75. 10.1038/s41556-020-0575-z. 32929201 10.1038/s41556-020-0575-z PMC7653546

[95] Rogers C, Fernandes-Alnemri T, Mayes L, Alnemri D, Cingolani G, Alnemri ES. Cleavage of DFNA5 by caspase-3 during apoptosis mediates progression to secondary necrotic/pyroptotic cell death. Nat Commun. 2017;8:14128. 10.1038/ncomms14128. 28045099 10.1038/ncomms14128 PMC5216131

[96] Wang Y, Gao W, Shi X, Ding J, Liu W, He H, et al. Chemotherapy drugs induce pyroptosis through caspase-3 cleavage of a gasdermin*.* Nature. 2017;547(7661) 10.1038/nature22393. 10.1038/nature22393 28459430

[97] Zhang Z, Zhang Y, Xia S, Kong Q, Li S, Liu X, et al. Gasdermin E suppresses tumour growth by activating anti-tumour immunity. Nature. 2020;579(7799):415–20. 10.1038/s41586-020-2071-9. 32188940 10.1038/s41586-020-2071-9 PMC7123794

[98] Xia S, Zhang Z, Magupalli VG, Pablo JL, Dong Y, Vora SM, et al. Gasdermin D pore structure reveals preferential release of mature interleukin-1. Nature. 2021;593(7860):607–11. 10.1038/s41586-021-03478-3. 33883744 10.1038/s41586-021-03478-3 PMC8588876

[99] Xia S. Biological mechanisms and therapeutic relevance of the gasdermin family. Mol Aspects Med. 2020;76: 100890. 10.1016/j.mam.2020.100890. 32800355 10.1016/j.mam.2020.100890 PMC7704569

[100] He K, Wan T, Wang D, Hu J, Zhou T, Tao W, et al. Gasdermin D licenses MHCII induction to maintain food tolerance in small intestine*.* Cell. 2023;186(14) 10.1016/j.cell.2023.05.027. 10.1016/j.cell.2023.05.027 37327784

[101] Deng M, Guo H, Tam JW, Johnson BM, Brickey WJ, New JS, et al. Platelet-activating factor (PAF) mediates NLRP3-NEK7 inflammasome induction independently of PAFR. J Exp Med. 2019;216(12):2838–53. 10.1084/jem.20190111. 31558613 10.1084/jem.20190111 PMC6888982

[102] Niu T, De Rosny C, Chautard S, Rey A, Patoli D, Groslambert M, et al. NLRP3 phosphorylation in its LRR domain critically regulates inflammasome assembly. Nat Commun. 2021;12(1):5862. 10.1038/s41467-021-26142-w. 34615873 10.1038/s41467-021-26142-w PMC8494922

[103] Fisch D, Bando H, Clough B, Hornung V, Yamamoto M, Shenoy A R, et al. Human GBP1 is a microbe-specific gatekeeper of macrophage apoptosis and pyroptosis*.* The EMBO Journal. 2019;38(13):e100926. 10.15252/embj.2018100926. 10.15252/embj.2018100926 PMC6600649 31268602

[104] Ratsimandresy RA, Chu LH, Khare S, de Almeida L, Gangopadhyay A, Indramohan M, et al. The PYRIN domain-only protein POP2 inhibits inflammasome priming and activation. Nat Commun. 2017;8:15556. 10.1038/ncomms15556. 28580931 10.1038/ncomms15556 PMC5465353

[105] Man S M, Karki R, Sasai M, Place D E, Kesavardhana S, Temirov J, et al. IRGB10 Liberates Bacterial Ligands for Sensing by the AIM2 and Caspase-11-NLRP3 Inflammasomes*.* Cell. 2016;167(2) 10.1016/j.cell.2016.09.012. 10.1016/j.cell.2016.09.012 PMC5074697 27693356

[106] Mathur A, Feng S, Hayward JA, Ngo C, Fox D, Atmosukarto II, et al. A multicomponent toxin from Bacillus cereus incites inflammation and shapes host outcome via the NLRP3 inflammasome. Nat Microbiol. 2019;4(2):362–74. 10.1038/s41564-018-0318-0. 30531979 10.1038/s41564-018-0318-0 PMC7685251

[107] Junqueira C, Crespo Â, Ranjbar S, de Lacerda LB, Lewandrowski M, Ingber J, et al. FcγR-mediated SARS-CoV-2 infection of monocytes activates inflammation. Nature. 2022;606(7914):576–84. 10.1038/s41586-022-04702-4. 35385861 10.1038/s41586-022-04702-4 PMC10071495

[108] Zhang C, Song J-W, Huang H-H, Fan X, Huang L, Deng J-N, et al. NLRP3 inflammasome induces CD4+ T cell loss in chronically HIV-1-infected patients*.* J Clin Invest. 2021;131(6) 10.1172/JCI138861. 10.1172/JCI138861 PMC7954596 33720048

[109] Doitsh G, Galloway NLK, Geng X, Yang Z, Monroe KM, Zepeda O, et al. Cell death by pyroptosis drives CD4 T-cell depletion in HIV-1 infection. Nature. 2014;505(7484):509–14. 10.1038/nature12940. 24356306 10.1038/nature12940 PMC4047036

[110] Monroe K M, Yang Z, Johnson J R, Geng X, tsh G, Krogan N J, et al. IFI16 DNA sensor is required for death of lymphoid CD4 T cells abortively infected with HIV*.* Science (New York, N.Y.). 2014;343(6169):428–32. 10.1126/science.1243640. 10.1126/science.1243640 PMC3976200 24356113

[111] Sefik E, Qu R, Junqueira C, Kaffe E, Mirza H, Zhao J, et al. Inflammasome activation in infected macrophages drives COVID-19 pathology. Nature. 2022;606(7914):585–93. 10.1038/s41586-022-04802-1. 35483404 10.1038/s41586-022-04802-1 PMC9288243

[112] Beckwith KS, Beckwith MS, Ullmann S, Sætra RS, Kim H, Marstad A, et al. Plasma membrane damage causes NLRP3 activation and pyroptosis during Mycobacterium tuberculosis infection. Nat Commun. 2020;11(1):2270. 10.1038/s41467-020-16143-6. 32385301 10.1038/s41467-020-16143-6 PMC7210277

[113] Kasper L, König A, Koenig P-A, Gresnigt MS, Westman J, Drummond RA, et al. The fungal peptide toxin Candidalysin activates the NLRP3 inflammasome and causes cytolysis in mononuclear phagocytes. Nat Commun. 2018;9(1):4260. 10.1038/s41467-018-06607-1. 30323213 10.1038/s41467-018-06607-1 PMC6189146

[114] Li Y, Shen Y, Jin K, Wen Z, Cao W, Wu B, et al. The DNA Repair Nuclease MRE11A Functions as a Mitochondrial Protector and Prevents T Cell Pyroptosis and Tissue Inflammation*.* Cell Metabolism. 2019;30(3) 10.1016/j.cmet.2019.06.016. 10.1016/j.cmet.2019.06.016 PMC7093039 31327667

[115] Yang X, Cheng X, Tang Y, Qiu X, Wang Y, Kang H, et al. Bacterial Endotoxin Activates the Coagulation Cascade through Gasdermin D-Dependent Phosphatidylserine Exposure*.* Immunity. 2019;51(6) 10.1016/j.immuni.2019.11.005. 10.1016/j.immuni.2019.11.005 31836429

[116] Zhu S, Ding S, Wang P, Wei Z, Pan W, Palm NW, et al. Nlrp9b inflammasome restricts rotavirus infection in intestinal epithelial cells. Nature. 2017;546(7660):667–70. 10.1038/nature22967. 28636595 10.1038/nature22967 PMC5787375

[117] Rauch I, Deets KA, Ji DX, von Moltke J, Tenthorey JL, Lee AY, et al. NAIP-NLRC4 Inflammasomes Coordinate Intestinal Epithelial Cell Expulsion with Eicosanoid and IL-18 Release via Activation of Caspase-1 and -8. Immunity. 2017;46(4):649–59. 10.1016/j.immuni.2017.03.016. 28410991 10.1016/j.immuni.2017.03.016 PMC5476318

[118] Karki R, Lee E, Place D, Samir P, Mavuluri J, Sharma B R, et al. IRF8 Regulates Transcription of Naips for NLRC4 Inflammasome Activation*.* Cell. 2018;173(4) 10.1016/j.cell.2018.02.055. 10.1016/j.cell.2018.02.055 PMC5935577 29576451

[119] Miao EA, Leaf IA, Treuting PM, Mao DP, Dors M, Sarkar A, et al. Caspase-1-induced pyroptosis is an innate immune effector mechanism against intracellular bacteria. Nat Immunol. 2010;11(12):1136–42. 10.1038/ni.1960. 21057511 10.1038/ni.1960 PMC3058225

[120] Qu Y, Misaghi S, Newton K, Maltzman A, Izrael-Tomasevic A, Arnott D, et al. NLRP3 recruitment by NLRC4 during Salmonella infection. J Exp Med. 2016;213(6):877–85. 10.1084/jem.20132234. 27139490 10.1084/jem.20132234 PMC4886354

[121] von Moltke J, Trinidad NJ, Moayeri M, Kintzer AF, Wang SB, van Rooijen N, et al. Rapid induction of inflammatory lipid mediators by the inflammasome in vivo. Nature. 2012;490(7418):107–11. 10.1038/nature11351. 22902502 10.1038/nature11351 PMC3465483

[122] Meunier E, Wallet P, Dreier RF, Costanzo S, Anton L, Rühl S, et al. Guanylate-binding proteins promote activation of the AIM2 inflammasome during infection with Francisella novicida. Nat Immunol. 2015;16(5):476–84. 10.1038/ni.3119. 25774716 10.1038/ni.3119 PMC4568307

[123] Rathinam VAK, Jiang Z, Waggoner SN, Sharma S, Cole LE, Waggoner L, et al. The AIM2 inflammasome is essential for host defense against cytosolic bacteria and DNA viruses. Nat Immunol. 2010;11(5):395–402. 10.1038/ni.1864. 20351692 10.1038/ni.1864 PMC2887480

[124] Wang L-Q, Liu T, Yang S, Sun L, Zhao Z-Y, Li L-Y, et al. Perfluoroalkyl substance pollutants activate the innate immune system through the AIM2 inflammasome. Nat Commun. 2021;12(1):2915. 10.1038/s41467-021-23201-0. 34006824 10.1038/s41467-021-23201-0 PMC8131593

[125] Xu H, Yang J, Gao W, Li L, Li P, Zhang L, et al. Innate immune sensing of bacterial modifications of Rho GTPases by the Pyrin inflammasome. Nature. 2014;513(7517):237–41. 10.1038/nature13449. 24919149 10.1038/nature13449

[126] Chung LK, Park YH, Zheng Y, Brodsky IE, Hearing P, Kastner DL, et al. The Yersinia Virulence Factor YopM Hijacks Host Kinases to Inhibit Type III Effector-Triggered Activation of the Pyrin Inflammasome. Cell Host Microbe. 2016;20(3):296–306. 10.1016/j.chom.2016.07.018. 27569559 10.1016/j.chom.2016.07.018 PMC5025386

[127] Sharif H, Hollingsworth L R, Griswold A R, Hsiao J C, Wang Q, Bachovchin D A, et al. Dipeptidyl peptidase 9 sets a threshold for CARD8 inflammasome formation by sequestering its active C-terminal fragment*.* Immunity. 2021;54(7) 10.1016/j.immuni.2021.04.024. 10.1016/j.immuni.2021.04.024 PMC8423358 34019797

[128] Wang Q, Gao H, Clark K M, Mugisha C S, Davis K, Tang J P, et al. CARD8 is an inflammasome sensor for HIV-1 protease activity*.* Science (New York, N.Y.). 2021;371(6535) 10.1126/science.abe1707. 10.1126/science.abe1707 PMC8029496 33542150

[129] Linder A, Bauernfried S, Cheng Y, Albanese M, Jung C, Keppler O T, et al. CARD8 inflammasome activation triggers pyroptosis in human T cells*.* EMBO J. 2020;39(19):e105071. 10.15252/embj.2020105071. 10.15252/embj.2020105071 PMC7527815 32840892

[130] Khare S, Dorfleutner A, Bryan NB, Yun C, Radian AD, de Almeida L, et al. An NLRP7-containing inflammasome mediates recognition of microbial lipopeptides in human macrophages. Immunity. 2012;36(3):464–76. 10.1016/j.immuni.2012.02.001. 22361007 10.1016/j.immuni.2012.02.001 PMC3315380

[131] Robinson K S, Teo D E T, Tan K S, Toh G A, Ong H H, Lim C K, et al. Enteroviral 3C protease activates the human NLRP1 inflammasome in airway epithelia*.* Science. 2020;370(6521) 10.1126/science.aay2002. 10.1126/science.aay2002 33093214

[132] Chui A J, Okondo M C, Rao S D, Gai K, Griswold A R, Johnson D C, et al. N-terminal degradation activates the NLRP1B inflammasome*.* Science (New York, N.Y.). 2019;364(6435):82–85. 10.1126/science.aau1208. 10.1126/science.aau1208 PMC6610862 30872531

[133] Van Opdenbosch N, Gurung P, Vande Walle L, Fossoul A, Kanneganti T-D, Lamkanfi M. Activation of the NLRP1b inflammasome independently of ASC-mediated caspase-1 autoproteolysis and speck formation. Nat Commun. 2014;5:3209. 10.1038/ncomms4209. 24492532 10.1038/ncomms4209 PMC3926011

[134] Chen R, Zeng L, Zhu S, Liu J, Zeh H J, Kroemer G, et al. cAMP metabolism controls caspase-11 inflammasome activation and pyroptosis in sepsis*.* Science Advances. 2019;5(5):eaav5562. 10.1126/sciadv.aav5562. 10.1126/sciadv.aav5562 PMC6531004 31131320

[135] Russo AJ, Vasudevan SO, Méndez-Huergo SP, Kumari P, Menoret A, Duduskar S, et al. Intracellular immune sensing promotes inflammation via gasdermin D-driven release of a lectin alarmin. Nat Immunol. 2021;22(2):154–65. 10.1038/s41590-020-00844-7. 33398185 10.1038/s41590-020-00844-7 PMC8916041

[136] Yang D, He Y, Muñoz-Planillo R, Liu Q, Núñez G. Caspase-11 Requires the Pannexin-1 Channel and the Purinergic P2X7 Pore to Mediate Pyroptosis and Endotoxic Shock. Immunity. 2015;43(5):923–32. 10.1016/j.immuni.2015.10.009. 26572062 10.1016/j.immuni.2015.10.009 PMC4795157

[137] Havira M S, Ta A, Kumari P, Wang C, Russo A J, Ruan J, et al. Shiga toxin suppresses noncanonical inflammasome responses to cytosolic LPS*.* Sci Immunol. 2020;5(53) 10.1126/sciimmunol.abc0217. 10.1126/sciimmunol.abc0217 PMC7717664 33246946

[138] Napier BA, Brubaker SW, Sweeney TE, Monette P, Rothmeier GH, Gertsvolf NA, et al. Complement pathway amplifies caspase-11-dependent cell death and endotoxin-induced sepsis severity. J Exp Med. 2016;213(11):2365–82. 27697835 10.1084/jem.20160027 PMC5068231

[139] Aachoui Y, Leaf I A, Hagar J A, Fontana M F, Campos C G, Zak D E, et al. Caspase-11 protects against bacteria that escape the vacuole*.* Science (New York, N.Y.). 2013;339(6122):975–78. 10.1126/science.1230751. 10.1126/science.1230751 PMC3697099 23348507

[140] Chu LH, Indramohan M, Ratsimandresy RA, Gangopadhyay A, Morris EP, Monack DM, et al. The oxidized phospholipid oxPAPC protects from septic shock by targeting the non-canonical inflammasome in macrophages. Nat Commun. 2018;9(1):996. 10.1038/s41467-018-03409-3. 29520027 10.1038/s41467-018-03409-3 PMC5843631

[141] Hagar J A, Powell D A, Aachoui Y, Ernst R K, and Miao E A. Cytoplasmic LPS activates caspase-11: implications in TLR4-independent endotoxic shock*.* Science (New York, N.Y.). 2013;341(6151):1250–53. 10.1126/science.1240988. 10.1126/science.1240988 PMC3931427 24031018

[142] Muendlein H I, Jetton D, Connolly W M, Eidell K P, Magri Z, Smirnova I, et al. cFLIPL protects macrophages from LPS-induced pyroptosis via inhibition of complex II formation*.* Science (New York, N.Y.). 2020;367(6484):1379–84. 10.1126/science.aay3878. 10.1126/science.aay3878 PMC7375259 32193329

[143] Demarco B, Grayczyk J P, Bjanes E, Le Roy D, Tonnus W, Assenmacher C-A, et al. Caspase-8-dependent gasdermin D cleavage promotes antimicrobial defense but confers susceptibility to TNF-induced lethality*.* Science Advances. 2020;6(47) 10.1126/sciadv.abc3465. 10.1126/sciadv.abc3465 PMC7673803 33208362

[144] Gaidt MM, Ebert TS, Chauhan D, Schmidt T, Schmid-Burgk JL, Rapino F, et al. Human Monocytes Engage an Alternative Inflammasome Pathway. Immunity. 2016;44(4):833–46. 10.1016/j.immuni.2016.01.012. 27037191 10.1016/j.immuni.2016.01.012

[145] Chen K W, Monteleone M, Boucher D, Sollberger G, Ramnath D, Condon N D, et al. Noncanonical inflammasome signaling elicits gasdermin D-dependent neutrophil extracellular traps*.* Sci Immunol. 2018;3(26) 10.1126/sciimmunol.aar6676. 10.1126/sciimmunol.aar6676 30143554

[146] Sollberger G, Choidas A, Burn G L, Habenberger P, Di Lucrezia R, Kordes S, et al. Gasdermin D plays a vital role in the generation of neutrophil extracellular traps*.* Sci Immunol. 2018;3(26) 10.1126/sciimmunol.aar6689. 10.1126/sciimmunol.aar6689 30143555

[147] Vats R, Kaminski TW, Brzoska T, Leech JA, Tutuncuoglu E, Katoch O, et al. Liver-to-lung microembolic NETs promote gasdermin D-dependent inflammatory lung injury in sickle cell disease. Blood. 2022;140(9):1020–37. 10.1182/blood.2021014552. 35737916 10.1182/blood.2021014552 PMC9437711

[148] Silva CMS, Wanderley CWS, Veras FP, Sonego F, Nascimento DC, Gonçalves AV, et al. Gasdermin D inhibition prevents multiple organ dysfunction during sepsis by blocking NET formation. Blood. 2021;138(25):2702–13. 10.1182/blood.2021011525. 34407544 10.1182/blood.2021011525 PMC8703366

[149] Burgener S S, Leborgne N G F, Snipas S J, Salvesen G S, Bird P I, and Benarafa C. Cathepsin G Inhibition by Serpinb1 and Serpinb6 Prevents Programmed Necrosis in Neutrophils and Monocytes and Reduces GSDMD-Driven Inflammation*.* Cell Rep. 2019;27(12) 10.1016/j.celrep.2019.05.065. 10.1016/j.celrep.2019.05.065 PMC7350907 31216481

[150] Clark KM, Kim JG, Wang Q, Gao H, Presti RM, Shan L. Chemical inhibition of DPP9 sensitizes the CARD8 inflammasome in HIV-1-infected cells. Nat Chem Biol. 2023;19(4):431–9. 10.1038/s41589-022-01182-5. 36357533 10.1038/s41589-022-01182-5 PMC10065922

[151] Liao Y, Chen X, Miller-Little W, Wang H, Willard B, Bulek K, et al. The Ras GTPase-activating-like protein IQGAP1 bridges Gasdermin D to the ESCRT system to promote IL-1β release via exosomes*.* The EMBO Journal. 2023;42(1):e110780. 10.15252/embj.2022110780. 10.15252/embj.2022110780 PMC9811620 36373462

[152] Nozaki K, Maltez VI, Rayamajhi M, Tubbs AL, Mitchell JE, Lacey CA, et al. Caspase-7 activates ASM to repair gasdermin and perforin pores. Nature. 2022;606(7916):960–7. 10.1038/s41586-022-04825-8. 35705808 10.1038/s41586-022-04825-8 PMC9247046

[153] Chen W, Chen S, Yan C, Zhang Y, Zhang R, Chen M, et al. Allergen protease-activated stress granule assembly and gasdermin D fragmentation control interleukin-33 secretion. Nat Immunol. 2022;23(7):1021–30. 10.1038/s41590-022-01255-6. 35794369 10.1038/s41590-022-01255-6 PMC11345751

[154] Wei C, Jiang W, Wang R, Zhong H, He H, Gao X, et al. Brain endothelial GSDMD activation mediates inflammatory BBB breakdown. Nature. 2024;629(8013):893–900. 10.1038/s41586-024-07314-2. 38632402 10.1038/s41586-024-07314-2

[155] Huang L S, Hong Z, Wu W, Xiong S, Zhong M, Gao X, et al. mtDNA Activates cGAS Signaling and Suppresses the YAP-Mediated Endothelial Cell Proliferation Program to Promote Inflammatory Injury*.* Immunity. 2020;52(3) 10.1016/j.immuni.2020.02.002. 10.1016/j.immuni.2020.02.002 PMC7266657 32164878

[156] Weindel C G, Martinez E L, Zhao X, Mabry C J, Bell S L, Vail K J, et al. Mitochondrial ROS promotes susceptibility to infection via gasdermin D-mediated necroptosis*.* Cell. 2022;185(17) 10.1016/j.cell.2022.06.038. 10.1016/j.cell.2022.06.038 PMC9531054 35907404

[157] Miao R, Jiang C, Chang W Y, Zhang H, An J, Ho F, et al. Gasdermin D permeabilization of mitochondrial inner and outer membranes accelerates and enhances pyroptosis*.* Immunity. 2023;56(11) 10.1016/j.immuni.2023.10.004. 10.1016/j.immuni.2023.10.004 PMC10872579 37924812

[158] Evavold C L, Hafner-Bratkovič I, Devant P, D'Andrea J M, Ngwa E M, Boršić E, et al. Control of gasdermin D oligomerization and pyroptosis by the Ragulator-Rag-mTORC1 pathway*.* Cell. 2021;184(17) 10.1016/j.cell.2021.06.028. 10.1016/j.cell.2021.06.028 PMC8380731 34289345

[159] Zhang N, Zhang J, Yang Y, Shan H, Hou S, Fang H, et al. A palmitoylation-depalmitoylation relay spatiotemporally controls GSDMD activation in pyroptosis. Nat Cell Biol. 2024;26(5):757–69. 10.1038/s41556-024-01397-9. 38538834 10.1038/s41556-024-01397-9

[160] Balasubramanian A, Hsu A Y, Ghimire L, Tahir M, Devant P, Fontana P, et al. The palmitoylation of gasdermin D directs its membrane translocation and pore formation during pyroptosis*.* Sci Immunol. 2024;9(94):eadn1452. 10.1126/sciimmunol.adn1452. 10.1126/sciimmunol.adn1452 PMC11367861 38530158

[161] Jiang X, Zhang X, Cai X, Li N, Zheng H, Tang M, et al. NU6300 covalently reacts with cysteine-191 of gasdermin D to block its cleavage and palmitoylation*.* Science Advances. 2024;10(6):eadi9284. 10.1126/sciadv.adi9284. 10.1126/sciadv.adi9284 PMC10849585 38324683

[162] Du G, Healy LB, David L, Walker C, El-Baba TJ, Lutomski CA, et al. ROS-dependent S-palmitoylation activates cleaved and intact gasdermin D. Nature. 2024;630(8016):437–46. 10.1038/s41586-024-07373-5. 38599239 10.1038/s41586-024-07373-5 PMC11283288

[163] Li Y, Pu D, Huang J, Zhang Y, Yin H. Protein phosphatase 1 regulates phosphorylation of gasdermin D and pyroptosis. Chem Commun (Camb). 2022;58(85):11965–8. 10.1039/d2cc03590a. 36205355 10.1039/d2cc03590a

[164] Michelucci A, Cordes T, Ghelfi J, Pailot A, Reiling N, Goldmann O, et al. Immune-responsive gene 1 protein links metabolism to immunity by catalyzing itaconic acid production. Proc Natl Acad Sci U S A. 2013;110(19):7820–5. 10.1073/pnas.1218599110. 23610393 10.1073/pnas.1218599110 PMC3651434

[165] Bambouskova M, Potuckova L, Paulenda T, Kerndl M, Mogilenko DA, Lizotte K, et al. Itaconate confers tolerance to late NLRP3 inflammasome activation. Cell Rep. 2021;34(10): 108756. 10.1016/j.celrep.2021.108756. 33691097 10.1016/j.celrep.2021.108756 PMC8039864

[166] Devant P, Boršić E, Ngwa EM, Xiao H, Chouchani ET, Thiagarajah JR, et al. Gasdermin D pore-forming activity is redox-sensitive. Cell Rep. 2023;42(1): 112008. 10.1016/j.celrep.2023.112008. 36662620 10.1016/j.celrep.2023.112008 PMC9947919

[167] Evavold C L, Ruan J, Tan Y, Xia S, Wu H, and Kagan J C. The Pore-Forming Protein Gasdermin D Regulates Interleukin-1 Secretion from Living Macrophages*.* Immunity. 2018;48(1) 10.1016/j.immuni.2017.11.013. 10.1016/j.immuni.2017.11.013 PMC5773350 29195811

[168] Zhang J, Yu Q, Jiang D, Yu K, Yu W, Chi Z, et al. Epithelial Gasdermin D shapes the host-microbial interface by driving mucus layer formation*.* Sci Immunol. 2022;7(68):eabk2092. 10.1126/sciimmunol.abk2092. 10.1126/sciimmunol.abk2092 35119941

[169] Volchuk A, Ye A, Chi L, Steinberg BE, Goldenberg NM. Indirect regulation of HMGB1 release by gasdermin D. Nat Commun. 2020;11(1):4561. 10.1038/s41467-020-18443-3. 32917873 10.1038/s41467-020-18443-3 PMC7486936

[170] Chi Z, Chen S, Yang D, Cui W, Lu Y, Wang Z, et al. Gasdermin D-mediated metabolic crosstalk promotes tissue repair. Nature. 2024;634(8036):1168–77. 10.1038/s41586-024-08022-7. 39260418 10.1038/s41586-024-08022-7

[171] Kayagaki N and Dixit V M. Rescue from a fiery death: A therapeutic endeavor*.* Science (New York, N.Y.). 2019;366(6466):688–89. 10.1126/science.aaw1177. 10.1126/science.aaw1177 31699924

[172] Degen M, Santos JC, Pluhackova K, Cebrero G, Ramos S, Jankevicius G, et al. Structural basis of NINJ1-mediated plasma membrane rupture in cell death. Nature. 2023;618(7967):1065–71. 10.1038/s41586-023-05991-z. 37198476 10.1038/s41586-023-05991-z PMC10307626

[173] Kayagaki N, Kornfeld OS, Lee BL, Stowe IB, O’Rourke K, Li Q, et al. NINJ1 mediates plasma membrane rupture during lytic cell death. Nature. 2021;591(7848):131–6. 10.1038/s41586-021-03218-7. 33472215 10.1038/s41586-021-03218-7

[174] David L, Borges J P, Hollingsworth L R, Volchuk A, Jansen I, Garlick E, et al. NINJ1 mediates plasma membrane rupture by cutting and releasing membrane disks*.* Cell. 2024;187(9) 10.1016/j.cell.2024.03.008. 10.1016/j.cell.2024.03.008 PMC11055670 38614101

[175] Kayagaki N, Stowe IB, Alegre K, Deshpande I, Wu S, Lin Z, et al. Inhibiting membrane rupture with NINJ1 antibodies limits tissue injury. Nature. 2023;618(7967):1072–7. 10.1038/s41586-023-06191-5. 37196676 10.1038/s41586-023-06191-5 PMC10307625

[176] Rühl S, Shkarina K, Demarco B, Heilig R, Santos J C, and Broz P. ESCRT-dependent membrane repair negatively regulates pyroptosis downstream of GSDMD activation*.* Science (New York, N.Y.). 2018;362(6417):956–60. 10.1126/science.aar7607. 10.1126/science.aar7607 30467171

[177] Li Z, Mo F, Wang Y, Li W, Chen Y, Liu J, et al. Enhancing Gasdermin-induced tumor pyroptosis through preventing ESCRT-dependent cell membrane repair augments antitumor immune response. Nat Commun. 2022;13(1):6321. 10.1038/s41467-022-34036-8. 36280674 10.1038/s41467-022-34036-8 PMC9592600

[178] Baatarjav C, Komada T, Karasawa T, Yamada N, Sampilvanjil A, Matsumura T, et al. dsDNA-induced AIM2 pyroptosis halts aberrant inflammation during rhabdomyolysis-induced acute kidney injury. Cell Death Differ. 2022;29(12):2487–502. 10.1038/s41418-022-01033-9. 35739254 10.1038/s41418-022-01033-9 PMC9750976

[179] Wang Y, Zhu X, Yuan S, Wen S, Liu X, Wang C, et al. TLR4/NF-κB Signaling Induces GSDMD-Related Pyroptosis in Tubular Cells in Diabetic Kidney Disease. Front Endocrinol (Lausanne). 2019;10:603. 10.3389/fendo.2019.00603. 31608008 10.3389/fendo.2019.00603 PMC6761221

[180] Nasrabady SE, Rizvi B, Goldman JE, Brickman AM. White matter changes in Alzheimer’s disease: a focus on myelin and oligodendrocytes. Acta Neuropathol Commun. 2018;6(1):22. 10.1186/s40478-018-0515-3. 29499767 10.1186/s40478-018-0515-3 PMC5834839

[181] Sun Y, Rong X, Li D, Jiang Y, Lu Y, Ji Y. Down-regulation of CRTAC1 attenuates UVB-induced pyroptosis in HLECs through inhibiting ROS production. Biochem Biophys Res Commun. 2020;532(1):159–65. 10.1016/j.bbrc.2020.07.028. 32838966 10.1016/j.bbrc.2020.07.028

[182] Method of the Year 2019: Single-cell multimodal omics*.* Nature Methods. 2020;17(1):1. 10.1038/s41592-019-0703-5. 10.1038/s41592-019-0703-5 31907477

[183] Xin J, Bao B, Liu J, Ma Z, Zhang M, Bi H, et al. Crosstalk between Myopia and Inflammation: A Mini Review. Int J Med Sci. 2024;21(9):1589–603. 10.7150/ijms.94826. 39006849 10.7150/ijms.94826 PMC11241089

[184] Malhotra S, Hayes D, and Wozniak D J. Cystic Fibrosis and Pseudomonas aeruginosa: the Host-Microbe Interface*.* Clin Microbiol Rev. 2019;32(3) 10.1128/CMR.00138-18. 10.1128/CMR.00138-18 PMC6589863 31142499

[185] Lee S, Nakahira K, Dalli J, Siempos II, Norris PC, Colas RA, et al. NLRP3 Inflammasome Deficiency Protects against Microbial Sepsis via Increased Lipoxin B4 Synthesis. Am J Respir Crit Care Med. 2017;196(6):713–26. 10.1164/rccm.201604-0892OC. 28245134 10.1164/rccm.201604-0892OC PMC5620673

[186] Jing W, Pilato J L, Kay C, Feng S, Tuipulotu D E, Mathur A, et al. Clostridium septicum α-toxin activates the NLRP3 inflammasome by engaging GPI-anchored proteins*.* Sci Immunol. 2022;7(71):eabm1803. 10.1126/sciimmunol.abm1803. 10.1126/sciimmunol.abm1803 35594341

[187] Valderrama JA, Riestra AM, Gao NJ, LaRock CN, Gupta N, Ali SR, et al. Group A streptococcal M protein activates the NLRP3 inflammasome. Nat Microbiol. 2017;2(10):1425–34. 10.1038/s41564-017-0005-6. 28784982 10.1038/s41564-017-0005-6 PMC5750061

[188] Cohen T S and Prince A S. Activation of inflammasome signaling mediates pathology of acute P. aeruginosa pneumonia*.* J Clin Invest. 2013;123(4):1630–37. 10.1172/JCI66142. 10.1172/JCI66142 PMC3613922 23478406

[189] Basiorka AA, McGraw KL, Eksioglu EA, Chen X, Johnson J, Zhang L, et al. The NLRP3 inflammasome functions as a driver of the myelodysplastic syndrome phenotype. Blood. 2016;128(25):2960–75. 10.1182/blood-2016-07-730556. 27737891 10.1182/blood-2016-07-730556 PMC5179338

[190] Huang X, Feng Z, Jiang Y, Li J, Xiang Q, Guo S, et al. VSIG4 mediates transcriptional inhibition of Nlrp3 and Il-1β in macrophages*.* Science Advances. 2019;5(1):eaau7426. 10.1126/sciadv.aau7426. 10.1126/sciadv.aau7426 PMC6326752 30662948

[191] Brydges SD, Broderick L, McGeough MD, Pena CA, Mueller JL, Hoffman HM. Divergence of IL-1, IL-18, and cell death in NLRP3 inflammasomopathies. J Clin Invest. 2013;123(11):4695–705. 24084736 10.1172/JCI71543 PMC3809806

[192] Magnotti F, Lefeuvre L, Benezech S, Malsot T, Waeckel L, Martin A, et al. Pyrin dephosphorylation is sufficient to trigger inflammasome activation in familial Mediterranean fever patients*.* EMBO Mol Med. 2019;11(11):e10547. 10.15252/emmm.201910547. 10.15252/emmm.201910547 PMC6835204 31589380

[193] Magnotti F, Malsot T, Georgin-Lavialle S, Abbas F, Martin A, Belot A, et al. Fast diagnostic test for familial Mediterranean fever based on a kinase inhibitor. Ann Rheum Dis. 2021;80(1):128–32. 10.1136/annrheumdis-2020-218366. 33037005 10.1136/annrheumdis-2020-218366

[194] Zhu H, Guan Y, Wang W, Liu X, Wang S, Zheng R, et al. Reniformin A suppresses non-small cell lung cancer progression by inducing TLR4/NLRP3/caspase-1/GSDMD-dependent pyroptosis. Int Immunopharmacol. 2024;133: 112068. 10.1016/j.intimp.2024.112068. 38626545 10.1016/j.intimp.2024.112068

[195] Yuan R, Zhao W, Wang Q-Q, He J, Han S, Gao H, et al. Cucurbitacin B inhibits non-small cell lung cancer in vivo and in vitro by triggering TLR4/NLRP3/GSDMD-dependent pyroptosis. Pharmacol Res. 2021;170: 105748. 10.1016/j.phrs.2021.105748. 34217831 10.1016/j.phrs.2021.105748

[196] Ding B, Chen H, Tan J, Meng Q, Zheng P, Ma P a, et al. ZIF-8 Nanoparticles Evoke Pyroptosis for High-Efficiency Cancer Immunotherapy*.* Angewandte Chemie (International Ed. In English). 2023;62(10):e202215307. 10.1002/anie.202215307. 10.1002/anie.202215307 36629270

[197] Dai J, Qu T, Yin D, Cui Y, Zhang C, Zhang E, et al. LncRNA LINC00969 promotes acquired gefitinib resistance by epigenetically suppressing of NLRP3 at transcriptional and posttranscriptional levels to inhibit pyroptosis in lung cancer. Cell Death Dis. 2023;14(5):312. 10.1038/s41419-023-05840-x. 37156816 10.1038/s41419-023-05840-x PMC10167249

[198] Yang J, Liu S, Li Y, Fan Z, Meng Y, Zhou B, et al. FABP4 in macrophages facilitates obesity-associated pancreatic cancer progression via the NLRP3/IL-1β axis. Cancer Lett. 2023;575: 216403. 10.1016/j.canlet.2023.216403. 37741433 10.1016/j.canlet.2023.216403

[199] Li M-Q, He Y-Q, Zhang M-N, Tang W, Tan Y, Cheng Y, et al. Dronedarone hydrochloride (DH) induces pancreatic cancer cell death by triggering mtDNA-mediated pyroptosis. Cell Death Dis. 2024;15(10):725. 10.1038/s41419-024-07102-w. 39358349 10.1038/s41419-024-07102-w PMC11447222

[200] Wang X, Ding B, Liu W, Qi L, Li J, Zheng X, et al. Dual Starvations Induce Pyroptosis for Orthotopic Pancreatic Cancer Therapy through Simultaneous Deprivation of Glucose and Glutamine. J Am Chem Soc. 2024;146(26):17854–65. 10.1021/jacs.4c03478. 38776361 10.1021/jacs.4c03478

[201] Lv T, Xiong X, Yan W, Liu M, Xu H, and He Q. Targeting of GSDMD sensitizes HCC to anti-PD-1 by activating cGAS pathway and downregulating PD-L1 expression*.* Journal For Immunotherapy of Cancer. 2022;10(6) 10.1136/jitc-2022-004763. 10.1136/jitc-2022-004763 PMC9189836 35688553

[202] Xu L, Peng F, Luo Q, Ding Y, Yuan F, Zheng L, et al. IRE1α silences dsRNA to prevent taxane-induced pyroptosis in triple-negative breast cancer*.* Cell. 2024;187(25) 10.1016/j.cell.2024.09.032. 10.1016/j.cell.2024.09.032 PMC11645245 39419025

[203] Yan H, Luo B, Wu X, Guan F, Yu X, Zhao L, et al. Cisplatin Induces Pyroptosis via Activation of MEG3/NLRP3/caspase-1/GSDMD Pathway in Triple-Negative Breast Cancer. Int J Biol Sci. 2021;17(10):2606–21. 10.7150/ijbs.60292. 34326697 10.7150/ijbs.60292 PMC8315016

[204] Su P, Mao X, Ma J, Huang L, Yu L, Tang S, et al. ERRα promotes glycolytic metabolism and targets the NLRP3/caspase-1/GSDMD pathway to regulate pyroptosis in endometrial cancer. J Exp Clin Cancer Res. 2023;42(1):274. 10.1186/s13046-023-02834-7. 37864196 10.1186/s13046-023-02834-7 PMC10588109

[205] Ning H, Huang S, Lei Y, Zhi R, Yan H, Jin J, et al. Enhancer decommissioning by MLL4 ablation elicits dsRNA-interferon signaling and GSDMD-mediated pyroptosis to potentiate anti-tumor immunity. Nat Commun. 2022;13(1):6578. 10.1038/s41467-022-34253-1. 36323669 10.1038/s41467-022-34253-1 PMC9630274

[206] Xia Y, Huang P, Qian Y-Y, Wang Z, Jin N, Li X, et al. PARP inhibitors enhance antitumor immune responses by triggering pyroptosis via TNF-caspase 8-GSDMD/E axis in ovarian cancer*.* Journal For Immunotherapy of Cancer. 2024;12(10) 10.1136/jitc-2024-009032. 10.1136/jitc-2024-009032 PMC11459312 39366751

[207] Fontana P, Du G, Zhang Y, Zhang H, Vora S M, Hu J J, et al. Small-molecule GSDMD agonism in tumors stimulates antitumor immunity without toxicity*.* Cell. 2024;187(22) 10.1016/j.cell.2024.08.007. 10.1016/j.cell.2024.08.007 PMC11648675 39243763

[208] Zhu G, Xie Y, Wang J, Wang M, Qian Y, Sun Q, et al. Multifunctional Copper-Phenolic Nanopills Achieve Comprehensive Polyamines Depletion to Provoke Enhanced Pyroptosis and Cuproptosis for Cancer Immunotherapy*.* Advanced Materials (Deerfield Beach, Fla.). 2024;36(45):e2409066. 10.1002/adma.202409066. 10.1002/adma.202409066 39285820

[209] Xie W, Peng M, Liu Y, Zhang B, Yi L, Long Y. Simvastatin induces pyroptosis via ROS/caspase-1/GSDMD pathway in colon cancer. Cell Commun Signal. 2023;21(1):329. 10.1186/s12964-023-01359-y. 37974278 10.1186/s12964-023-01359-y PMC10652480

[210] Suh JJ, Pettinati HM, Kampman KM, O’Brien CP. The status of disulfiram: a half of a century later. J Clin Psychopharmacol. 2006;26(3):290–302. 16702894 10.1097/01.jcp.0000222512.25649.08

[211] Rathkey J K, Zhao J, Liu Z, Chen Y, Yang J, Kondolf H C, et al. Chemical disruption of the pyroptotic pore-forming protein gasdermin D inhibits inflammatory cell death and sepsis*.* Sci Immunol. 2018;3(26) 10.1126/sciimmunol.aat2738. 10.1126/sciimmunol.aat2738 PMC6462819 30143556

[212] Hu JJ, Liu X, Xia S, Zhang Z, Zhang Y, Zhao J, et al. FDA-approved disulfiram inhibits pyroptosis by blocking gasdermin D pore formation. Nat Immunol. 2020;21(7):736–45. 10.1038/s41590-020-0669-6. 32367036 10.1038/s41590-020-0669-6 PMC7316630

[213] Humphries F, Shmuel-Galia L, Ketelut-Carneiro N, Li S, Wang B, Nemmara VV, et al. Succination inactivates gasdermin D and blocks pyroptosis. Science. 2020;369(6511):1633–7. 10.1126/science.abb9818. 32820063 10.1126/science.abb9818 PMC8744141

[214] Li Y, Tu Z, Chen F, Yang X, Deng R, Su F, et al. Anti-inflammatory effect of Danhong injection through inhibition of GSDMD-mediated pyroptosis. Phytomedicine. 2023;113: 154743. 10.1016/j.phymed.2023.154743. 36893672 10.1016/j.phymed.2023.154743

[215] Chen Y, Luo R, Li J, Wang S, Ding J, Zhao K, et al. Intrinsic Radical Species Scavenging Activities of Tea Polyphenols Nanoparticles Block Pyroptosis in Endotoxin-Induced Sepsis. ACS Nano. 2022;16(2):2429–41. 10.1021/acsnano.1c08913. 35133795 10.1021/acsnano.1c08913

[216] Martín-Sánchez F, Diamond C, Zeitler M, Gomez AI, Baroja-Mazo A, Bagnall J, et al. Inflammasome-dependent IL-1β release depends upon membrane permeabilisation. Cell Death Differ. 2016;23(7):1219–31. 10.1038/cdd.2015.176. 26868913 10.1038/cdd.2015.176 PMC4946890

[217] Zhong L, Han J, Fan X, Huang Z, Su L, Cai X, et al. Novel GSDMD inhibitor GI-Y1 protects heart against pyroptosis and ischemia/reperfusion injury by blocking pyroptotic pore formation. Basic Res Cardiol. 2023;118(1):40. 10.1007/s00395-023-01010-4. 37782407 10.1007/s00395-023-01010-4

[218] Huo S, Zhao P, Shi Z, Zou M, Yang X, Warszawik E, et al. Mechanochemical bond scission for the activation of drugs. Nat Chem. 2021;13(2):131–9. 10.1038/s41557-020-00624-8. 33514936 10.1038/s41557-020-00624-8

[219] Su X, Wang W-J, Cao Q, Zhang H, Liu B, Ling Y, et al. A Carbonic Anhydrase IX (CAIX)-Anchored Rhenium(I) Photosensitizer Evokes Pyroptosis for Enhanced Anti-Tumor Immunity*.* Angewandte Chemie (International Ed. In English). 2022;61(8):e202115800. 10.1002/anie.202115800. 10.1002/anie.202115800 34842317

[220] Li J, Ding B, Tan J, Chen H, Meng Q, Li X, et al. Sodium Citrate Nanoparticles Induce Dual-Path Pyroptosis for Enhanced Antitumor Immunotherapy through Synergistic Ion Overload and Metabolic Disturbance. Nano Lett. 2023;23(21):10034–43. 10.1021/acs.nanolett.3c03382. 37903236 10.1021/acs.nanolett.3c03382

[221] Yang Y, Yang J, Zhu N, Qiu H, Feng W, Chen Y, et al. Tumor-targeting hydroxyapatite nanoparticles for remodeling tumor immune microenvironment (TIME) by activating mitoDNA-pyroptosis pathway in cancer. Journal of Nanobiotechnology. 2023;21(1):470. 10.1186/s12951-023-02231-4. 38062467 10.1186/s12951-023-02231-4 PMC10704647

[222] Song X, Huang H, Xia L, Jia W, Yang S, Wang C, et al. Engineering 2D Multienzyme-Mimicking Pyroptosis Inducers for Ultrasound-Augmented Catalytic Tumor Nanotherapy*.* Advanced Science (Weinheim, Baden-Wurttemberg, Germany). 2023;10(24):e2301279. 10.1002/advs.202301279. 10.1002/advs.202301279 PMC10460896 37350357

[223] Zhou H, Qian Q, Chen Q, Chen T, Wu C, Chen L, et al. Enhanced Mitochondrial Targeting and Inhibition of Pyroptosis with Multifunctional Metallopolyphenol Nanoparticles in Intervertebral Disc Degeneration. Small. 2024;20(13): e2308167. 10.1002/smll.202308167. 37953455 10.1002/smll.202308167

[224] Gao M, Chen J, Chen C, Xie M, Xie Q, Li W, et al. Nano-microflora Interaction Inducing Pulmonary Inflammation by Pyroptosis. Environ Sci Technol. 2024;58(20):8643–53. 10.1021/acs.est.4c00141. 38676641 10.1021/acs.est.4c00141

[225] Wei Y, Zhu M, Li S, Hong T, Guo X, Li Y, et al. Engineered Biomimetic Nanoplatform Protects the Myocardium Against Ischemia/Reperfusion Injury by Inhibiting Pyroptosis. ACS Appl Mater Interfaces. 2021;13(29):33756–66. 10.1021/acsami.1c03421. 34258997 10.1021/acsami.1c03421

[226] Liao Z, Liu H, Ma L, Lei J, Tong B, Li G, et al. Engineering Extracellular Vesicles Restore the Impaired Cellular Uptake and Attenuate Intervertebral Disc Degeneration. ACS Nano. 2021;15(9):14709–24. 10.1021/acsnano.1c04514. 34476937 10.1021/acsnano.1c04514

[227] Aghajanian H, Kimura T, Rurik JG, Hancock AS, Leibowitz MS, Li L, et al. Targeting cardiac fibrosis with engineered T cells. Nature. 2019;573(7774):430–3. 10.1038/s41586-019-1546-z. 31511695 10.1038/s41586-019-1546-z PMC6752964

[228] Rettig WJ, Garin-Chesa P, Beresford HR, Oettgen HF, Melamed MR, Old LJ. Cell-surface glycoproteins of human sarcomas: differential expression in normal and malignant tissues and cultured cells. Proc Natl Acad Sci U S A. 1988;85(9):3110–4. 2896356 10.1073/pnas.85.9.3110 PMC280153

[229] Niedermeyer J, Scanlan MJ, Garin-Chesa P, Daiber C, Fiebig HH, Old LJ, et al. Mouse fibroblast activation protein: molecular cloning, alternative splicing and expression in the reactive stroma of epithelial cancers. Int J Cancer. 1997;71(3):383–9. 9139873 10.1002/(sici)1097-0215(19970502)71:3<383::aid-ijc14>3.0.co;2-h

[230] Wang K, Sun Q, Zhong X, Zeng M, Zeng H, Shi X, et al. Structural Mechanism for GSDMD Targeting by Autoprocessed Caspases in Pyroptosis*.* Cell. 2020;180(5) 10.1016/j.cell.2020.02.002. 10.1016/j.cell.2020.02.002 32109412

[231] Kim H, Wang S Y, Kwak G, Yang Y, Kwon I C, and Kim S H. Exosome-Guided Phenotypic Switch of M1 to M2 Macrophages for Cutaneous Wound Healing*.* Advanced Science (Weinheim, Baden-Wurttemberg, Germany). 2019;6(20):1900513. 10.1002/advs.201900513. 10.1002/advs.201900513 PMC6794619 31637157

[232] Sreejit G, Abdel-Latif A, Athmanathan B, Annabathula R, Dhyani A, Noothi SK, et al. Neutrophil-Derived S100A8/A9 Amplify Granulopoiesis After Myocardial Infarction. Circulation. 2020;141(13):1080–94. 10.1161/CIRCULATIONAHA.119.043833. 31941367 10.1161/CIRCULATIONAHA.119.043833 PMC7122461

